# The Diagnostic Value of Capillary Refill Time for Detecting Serious Illness in Children: A Systematic Review and Meta-Analysis

**DOI:** 10.1371/journal.pone.0138155

**Published:** 2015-09-16

**Authors:** Susannah Fleming, Peter Gill, Caroline Jones, James A. Taylor, Ann Van den Bruel, Carl Heneghan, Nia Roberts, Matthew Thompson

**Affiliations:** 1 Nuffield Department of Primary Care Health Sciences, University of Oxford, Oxford, Oxfordshire, United Kingdom; 2 The Hospital for Sick Children, Department of Pediatrics, University of Toronto, Toronto, Ontario, Canada; 3 Child Health Institute, University of Washington, Seattle, Washington, United States of America; 4 Bodleian Health Care Libraries, University of Oxford, Oxford, Oxfordshire, United Kingdom; 5 Department of Family Medicine, University of Washington, Seattle, Washington, United States of America; Institute of Tropical Medicine (NEKKEN), Nagasaki University, JAPAN

## Abstract

**Importance:**

Capillary refill time (CRT) is widely recommended as part of the routine assessment of unwell children.

**Objective:**

To determine the diagnostic value of capillary refill time for a range of serious outcomes in children.

**Methods:**

We searched Medline, Embase and CINAHL from inception to June 2014. We included studies that measured both capillary refill time and a relevant clinical outcome such as mortality, dehydration, meningitis, or other serious illnesses in children aged up to 18 years of age. We screened 1,265 references, of which 24 papers were included in this review. Where sufficient studies were available, we conducted meta-analysis and constructed hierarchical summary ROC curves.

**Results:**

Meta-analysis on the relationship between capillary refill time and mortality resulted in sensitivity of 34.6% (95% CI 23.9 to 47.1%), specificity 92.3% (88.6 to 94.8%), positive likelihood ratio 4.49 (3.06 to 6.57), and negative likelihood ratio 0.71 (0.60 to 0.84). Studies of children attending Emergency Departments with vomiting and diarrhea showed that capillary refill time had specificity of 89 to 94% for identifying 5% dehydration, but sensitivity ranged from 0 to 94%. This level of heterogeneity precluded formal meta-analysis of this outcome. Meta-analysis was not possible for other outcomes due to insufficient data, but we found consistently high specificity for a range of outcomes including meningitis, sepsis, admission to hospital, hypoxia, severity of illness and dengue.

**Conclusions:**

Our results show that capillary refill time is a specific sign, indicating that it can be used as a “red-flag”: children with prolonged capillary refill time have a four-fold risk of dying compared to children with normal capillary refill time. The low sensitivity means that a normal capillary refill time should not reassure clinicians.

## Introduction

Identifying children who may have hemodynamic compromise or shock is a routine part of clinical assessment and a core component of Pediatric Advanced Life Support.[1–3] In most settings, this includes assessment of peripheral perfusion such as capillary refill time (CRT) as well as measurement of blood pressure to assess cardiovascular status. However, blood pressure is maintained in children in the early stages of shock to a greater extent than in adults, to the expense of reduced peripheral perfusion and raised heart rate, so may be a less valuable measure particularly in triage or other frontline healthcare settings.[4, 5]

In a recent systematic review of the validity and reliability of CRT,[6] we found that CRT is 2 seconds or less when measured on the finger in healthy children and older infants, but may extend to 4 seconds when measured on the chest or foot. In addition to the influence of the body site at which CRT is measured, changes in both ambient and skin temperature may have a significant effect on measured values. Current evidence also indicates that inter-observer reliability of CRT is highly variable, and may be related to the measurement method, with those made using stopwatches displaying lower inter-observer variability than simple counting.

Despite the evidence on variability in measurements and a lack of standardization, CRT is included as part of the routine or initial assessment of seriously ill or injured children in Pediatric Advanced Life Support and in a number of major international guidelines (Table 1).[1–3, 7–9] CRT is also frequently recommended in monitoring and guiding the treatment of children with severe sepsis or septic shock.[4, 5]

**Table 1:** Current guideline recommendations for CRT.

Guideline	Recommendations for CRT use
**Advanced Paediatric Life Support (APLS)[1]**	CRT assessment advised as part of primary circulation assessment.
	“…capillary refill should occur within 2–3 seconds. A slower refill time than this …. is a particularly useful sign in early septic shock… Poor capillary refill and differential pulse volumes are neither sensitive nor specific indicators of shock in infants and children. . .”
**Pediatric Advanced Life Support (PALS)[2]**	CRT assessment advised as part of primary circulation assessment.
	“Frequent causes of sluggish, delayed or prolonged capillary refill (a refill time >2 seconds) include dehydration, shock, and hypothermia. Shock can be present despite a normal capillary refill time. Children in “warm” septic shock may have excellent (ie, <2 seconds) capillary refill time.”
**WHO guidelines for the management of children with severe infection or severe malnutrition[3]**	“Emergency signs include: … signs of shock (capillary refill longer than 3 seconds; and fast weak pulse)…. Children with emergency signs require immediate treatment to avert death.”
	“If capillary refill is longer than 3 seconds, check the pulse.”
**ACCCM Guidelines for pediatric and neonatal septic shock[4]**	“The clinical diagnosis of septic shock is made in children who 1) have a suspected infection manifested by hypothermia or hyperthermia, and 2) have clinical signs of inadequate tissue perfusion including any of the following; … prolonged capillary refill >2 secs (cold shock), … or flash capillary refill (warm shock)”
	“Therapeutic End Points (Level III). Capillary refill < = 2 secs,…”
**International guidelines for severe sepsis and septic shock[5]**	“We suggest that the initial therapeutic endpoints of resuscitation of septic shock be capillary refill of < = 2 s…”
**NICE Guidelines on Feverish Illness in children[9]**	“Measure and record temperature, heart rate, respiratory rate and capillary refill time as part of the routine assessment of a child with fever.”
	“Recognize that a capillary refill time of 3 seconds or longer is an intermediate-risk group marker for serious illness (‘amber’ sign).”
	“Assess children with fever for signs of dehydration. Look for: prolonged capillary refill time…”
	“Consider meningococcal disease in any child with fever and a non-blanching rash, particularly if any of the following features are present: … a capillary refill time of 3 seconds or longer”
	“A prolonged CRT may be a sign of circulatory insufficiency (e.g. shock) or dehydration”
**NICE Guidelines on Bacterial meningitis and meningococcal septicemia[8]**	Capillary refill time more than 2 seconds defined as “more specific symptoms/signs” for meningococcal disease and meningococcal septicemia, but not for bacterial meningitis.
	“Signs of shock: Capillary refill time more than 2 seconds…”
	“In children and young people with suspected bacterial meningitis or meningococcal septicemia, undertake and record physiological observations of … perfusion (capillary refill) … at least hourly.”
	“The clinical features in a febrile child or young person with petechiae that are more likely to suggest meningococcal disease are … prolonged capillary refill time …”
	“A prolonged CRT may be a sign of circulatory insufficiency (such as shock) or dehydration.”

Prolonged CRT or decreased peripheral perfusion is cited as a red flag for serious illness in children.[10–13] However, to our knowledge there has not been a comprehensive and systematic assessment of the diagnostic value of CRT across a range of serious conditions in children of all ages in all settings. This systematic review assesses the current evidence for the diagnostic and prognostic value of CRT for identifying children with serious illnesses such as dehydration and meningitis, and to predict adverse outcomes such as death.

## Methods

A protocol was developed by the authors for this review. This has not been registered or published, but is provided as S1 Text.

### Search strategies

A detailed search strategy was developed in collaboration with an information specialist (NR) to identify papers on the diagnostic accuracy of CRT in children (see S2 Text for details). The search strategy was applied to three bibliographic databases Medline (OvidSP) [1946-present], Embase (OvidSP) [1974-present] and CINAHL (OvidSP) [1980-present]. Additional papers were identified from the reference lists of relevant papers and consultation with experts. Search date was 9 June 2014. Searches were not restricted by language or country of origin.

### Inclusion and exclusion criteria

We included papers which reported measurement of CRT by any method on a minimum of 20 subjects under the age of 18 years. We excluded papers that reported data only on subjects over the age of 18 years, or in which more than 50% of the children were neonates born prematurely (<35 weeks gestation). We also excluded studies in which more than 50% of subjects had significant pre-existing cardiorespiratory disease such as cardiac malformations. However, we included studies that reported these groups separately, allowing extraction of a group with at least 20 children who fulfilled the inclusion criteria.

Eligible studies measured and reported the relationship between CRT and a relevant clinical outcome, with no other restriction on study design or clinical setting. Acceptable outcomes included: mortality; final diagnosis of a specific serious illness (e.g. meningitis, dehydration, or pneumonia); admission to secondary care facilities; hypoxia; and measures of severity of illness or infection composed of groupings of other acceptable outcomes (e.g. where “severe illness” is defined by hypoxia or a specific list of final diagnoses). Studies performing multivariate analyses including CRT with other clinical and/or laboratory tests were not included in this review unless univariate results for CRT and a relevant outcome were also presented.

References were screened for inclusion by two reviewers using title and abstract, using full-text where necessary. Subsequently, four reviewers assessed selected studies for eligibility using the full text. Where necessary we attempted to contact authors for further information to clarify eligibility.

### Quality criteria

Quality assessment criteria were developed based on the QUADAS-2 checklist.[14] The criteria (S1 Table) assessed patient selection, performance of the index test (CRT), performance of the comparator, and aspects of timing and flow.

### Data extraction

Data from all included studies were extracted on a pre-specified form by one author and checked by a second author, with any disagreements resolved by consensus. We extracted data on patient characteristics and study design, country of origin, and method of measuring CRT. Measurements of CRT were considered to be prolonged based on the definitions used by the investigators for each included study. All relevant outcomes were identified and extracted. Where they were not reported, we calculated sensitivity, specificity, and likelihood ratios, including 95% confidence intervals, from the 2x2 tables. Where one or more cells of a 2x2 table was empty, we added 0.5 to each cell before calculating summary statistics.[15] Where insufficient data were reported to calculate outcome measures or reconstruct 2x2 tables, we contacted study authors to obtain further information. Study settings were categorized into high-income, upper-middle-income, lower-middle-income, and low-income countries based on their World Bank classification in July 2013.[16]

### Analysis

Results are reported descriptively, identifying and explaining differences between studies and any resulting heterogeneity. When comparable outcomes were reported by at least four studies and no apparent clinical heterogeneity was present, meta-analysis was performed using the bivariate method.[17] Based on the parameters from this analysis, a hierarchical summary receiver operative characteristic (ROC) curve was constructed. When data on more than one cut-off was available, only one was included in the meta-analysis to avoid inclusion of data on the same patient twice. Where necessary, we included the data corresponding to the cut-off which was used most frequently in other studies within the meta-analysis, in an attempt to reduce overall heterogeneity. Statistical heterogeneity was inspected visually on the ROC plot by checking the distance of the individual studies from the hierarchical summary ROC curve. When statistical heterogeneity was present, no pooled results were reported.

Analyses were carried out using R (version 3.0.1, The R Foundation for Statistical Computing), with Stata (version 11.2, Statacorp) additionally used to generate hierarchical summary ROC curves.

## Results

**Fig 1:**
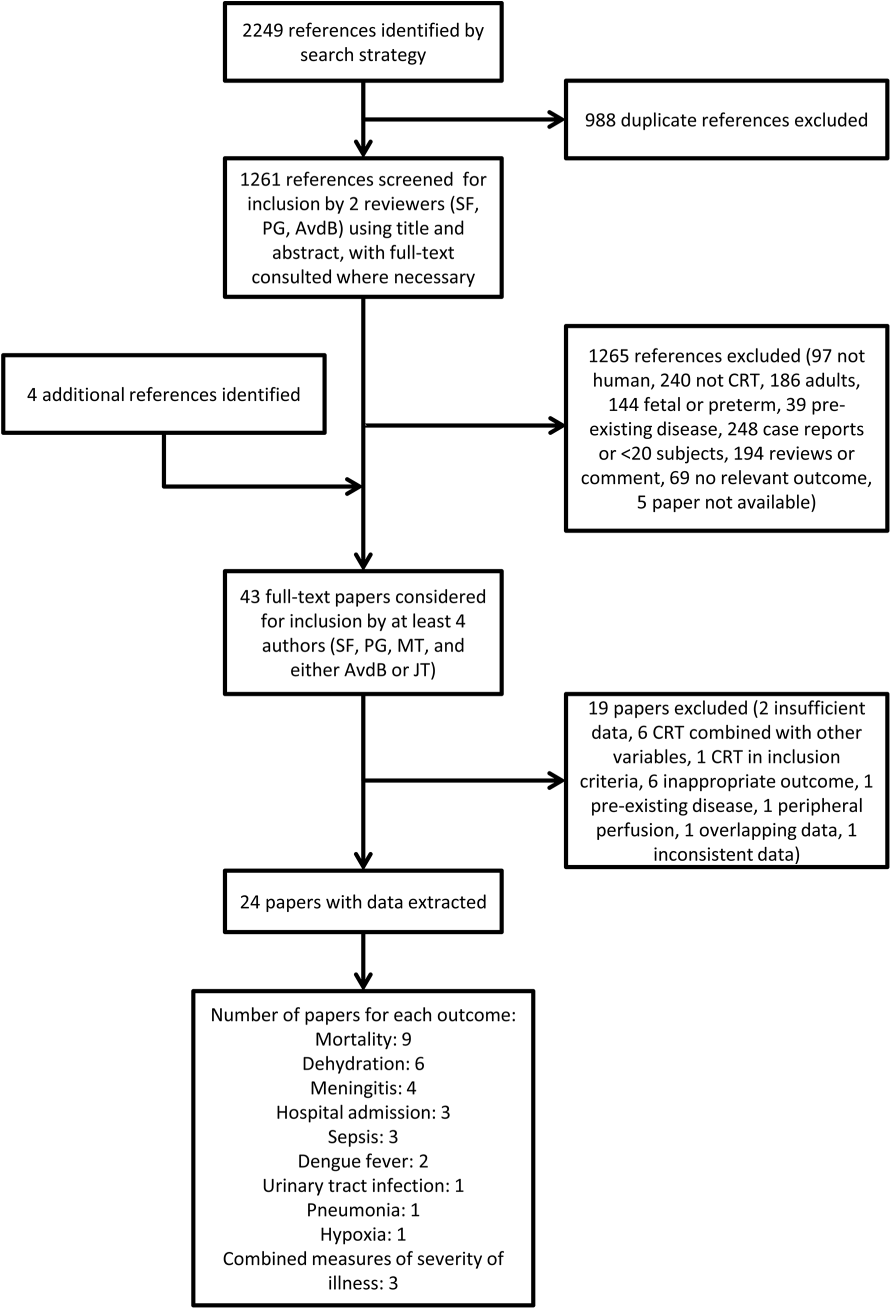
Flow chart for papers identified by the search strategy.

After removal of duplicates, we screened 1,265 references (Fig 1), and included 24 papers in this review, of which 13 reported studies carried out in high income countries, and 11 in low or middle income countries. Nine studies on 17,285 children assessed the relationship between CRT and mortality, and six studies on approximately 680 children assessed the relationship between CRT and dehydration, allowing meta-analyses on both outcomes. We also identified 11 papers on 41,287 children reporting other outcomes related to serious illness, including meningitis (four studies, n = 10,205), hospital admission (three studies, n = 17,742), sepsis (three studies, n = 19,923), dengue fever (two studies, n = 2,039), urinary tract infection (one study, n = 15,781), pneumonia (one study, n = 15,781), hypoxia (one study, n = 3,176), and combined measures of severity of illness (three studies, n = 8,555), with some studies reporting multiple outcomes. Full details of individual study characteristics are given in S2 Table.

**Fig 2:**
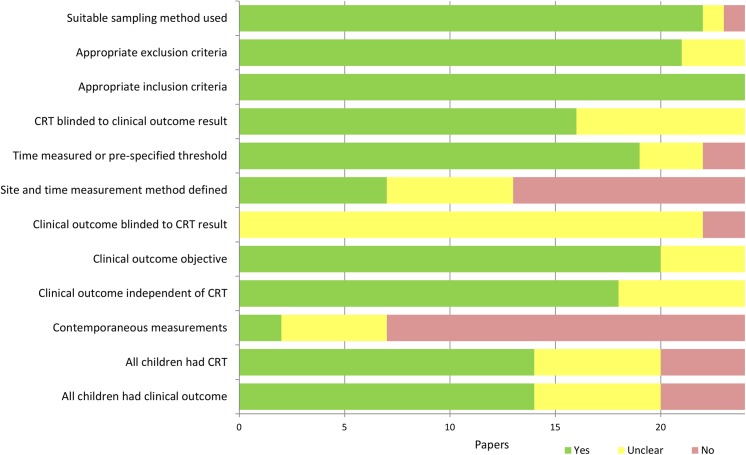
Summary of quality of included papers.

The quality assessment of the included papers is summarized in Fig 2, with details in S3 Table. Although performance on most of the quality criteria was good, reporting of the method of measurement of CRT was generally poor, with 11 papers reporting neither the site nor the methods of time measurement, and a further six reporting only one of these (noted as unclear reporting). Blinding was also poorly reported, particularly for the clinical outcomes. In many cases, blinding of CRT measurement could only be established by timing, in that the measurement was made before determination of the clinical outcome. Few studies had contemporaneous measurement of CRT and clinical outcome, although it should be noted that this was impractical or impossible for some outcomes, such as final clinical diagnosis, or mortality. Missing data were generally not reported leading to uncertainty regarding the representativeness of the sample.

### Predictive accuracy of CRT for mortality

We extracted 2x2 tables from seven of the studies on mortality, and data from the other two[18, 19] were obtained after communication with the authors (for details of extracted data, see S4 Table). The nine included studies provided data on 17,285 children, of whom 1,149 died. Eight of the studies were conducted in hospital settings in low or lower-middle income countries.

**Fig 3:**
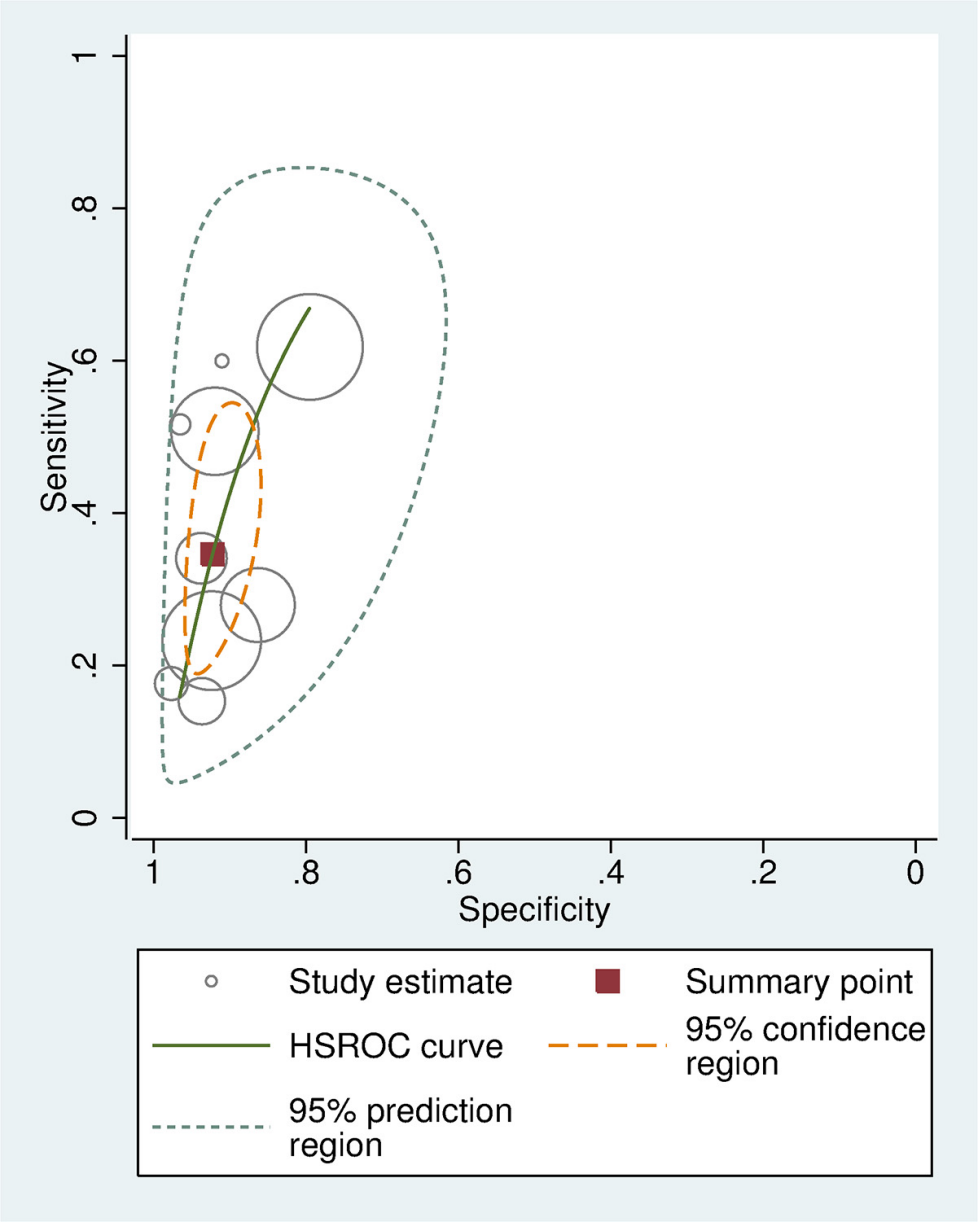
Hierarchical summary ROC curve showing diagnostic accuracy of CRT for predicting mortality.

In all nine studies, CRT had high specificity for predicting overall mortality (range of 80–98%). All but two showed low sensitivity: one reporting moderate sensitivity was a very small study with wide confidence intervals,[20] and the second recruited children in a different setting (pre-hospital emergency transport in a high income country).[21] We used data from the nine studies to calculate a hierarchical summary ROC (HSROC) curve, shown in green in Fig 3. The summary point calculated by the meta-analysis (shown as a red box in Fig 3) has a sensitivity of 34 6% (95% CI 23 9–47 1%), specificity 92 3% (88 6–94 8%), positive likelihood ratio 4 49 (3 06–6 57), and negative likelihood ratio 0 71 (0 60–0 84) for prolonged CRT as a predictor of mortality. A bivariate random effects model was used to estimate a 95% prediction region for the curve, and a 95% confidence region for the summary point.

**Fig 4:**
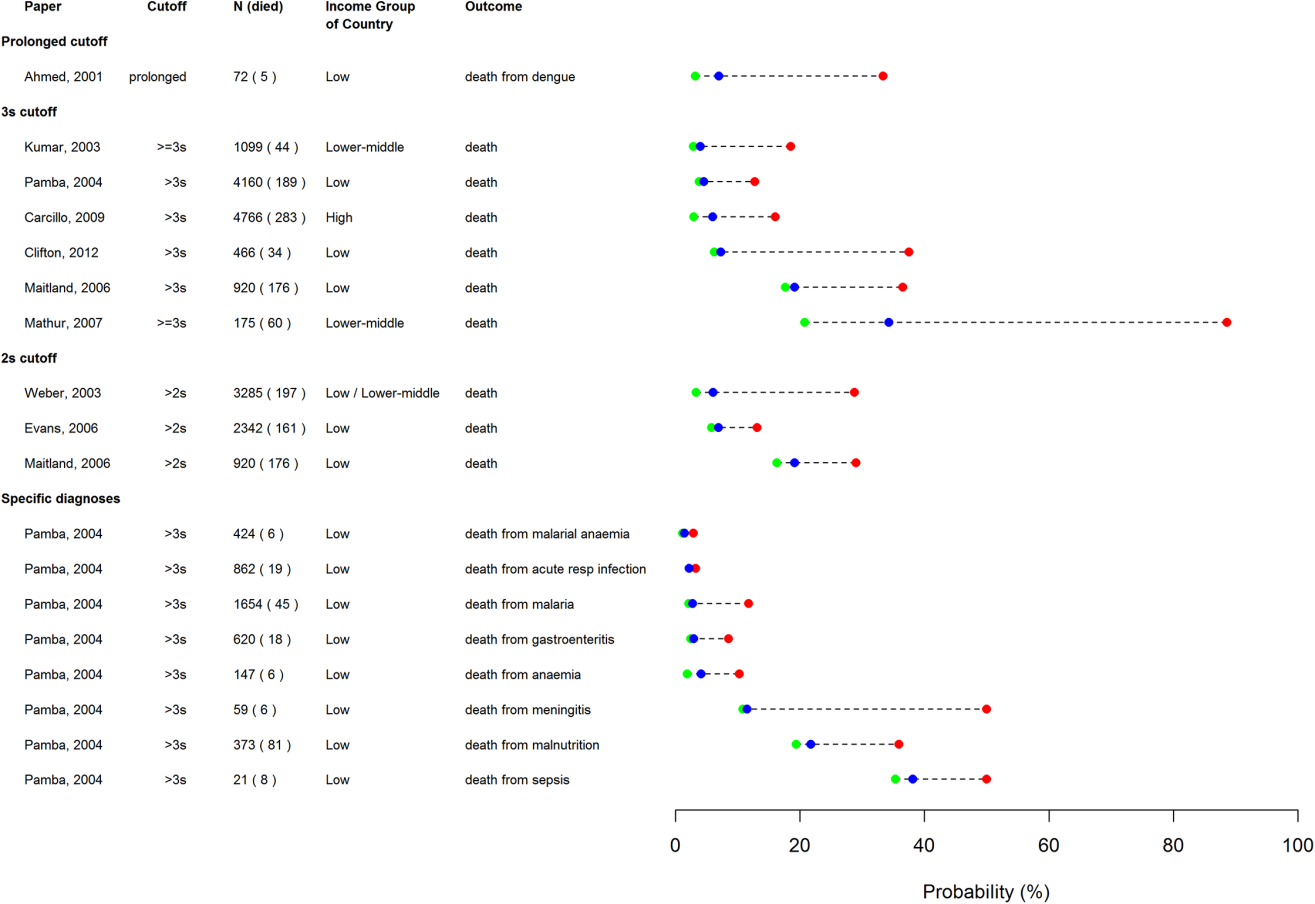
Pre/post test probability plot of prolonged CRT for predicting mortality. Markers show pre-test prevalence (blue), and post-test probabilities after positive (red) and negative (green) tests. Multiple results are shown for two studies: results are shown for two different cut-offs reported by Maitland et al,[22] and results from a subgroup analysis by Pamba and Maitland showing the performance for predicting death from different conditions[23].

In the dumbbell plot in Fig 4, the pre-test probability (prevalence) is shown in blue for each study, with the post-test probability after finding prolonged CRT (positive test) in red, and the post-test probability after finding normal CRT (negative test) in green. It can be seen that prolonged CRT consistently increases the post-test probability, whereas normal CRT does not greatly reduce it.

One large study[23] reported the mortality due to various causes with an abnormal CRT, as shown in the lower portion of the dumbbell plot in Fig 4. This indicates that CRT longer than 3 seconds is predictive of mortality from malaria (without severe anemia), malnutrition, meningitis, and sepsis, but not from acute respiratory infection or malarial anemia (Hb<50g/l).

### Diagnostic accuracy of CRT for dehydration

Of the six papers reporting the diagnostic accuracy of CRT for dehydration, recruitment overlapped in two[24, 25] (we selected the one with the larger cohort, more clearly defined measurement method, and more data on the outcome of interest for analysis.[25]) The number of children in the five included studies is 499, of whom 147 had ≥5% dehydration (see S5 Table).

**Fig 5:**
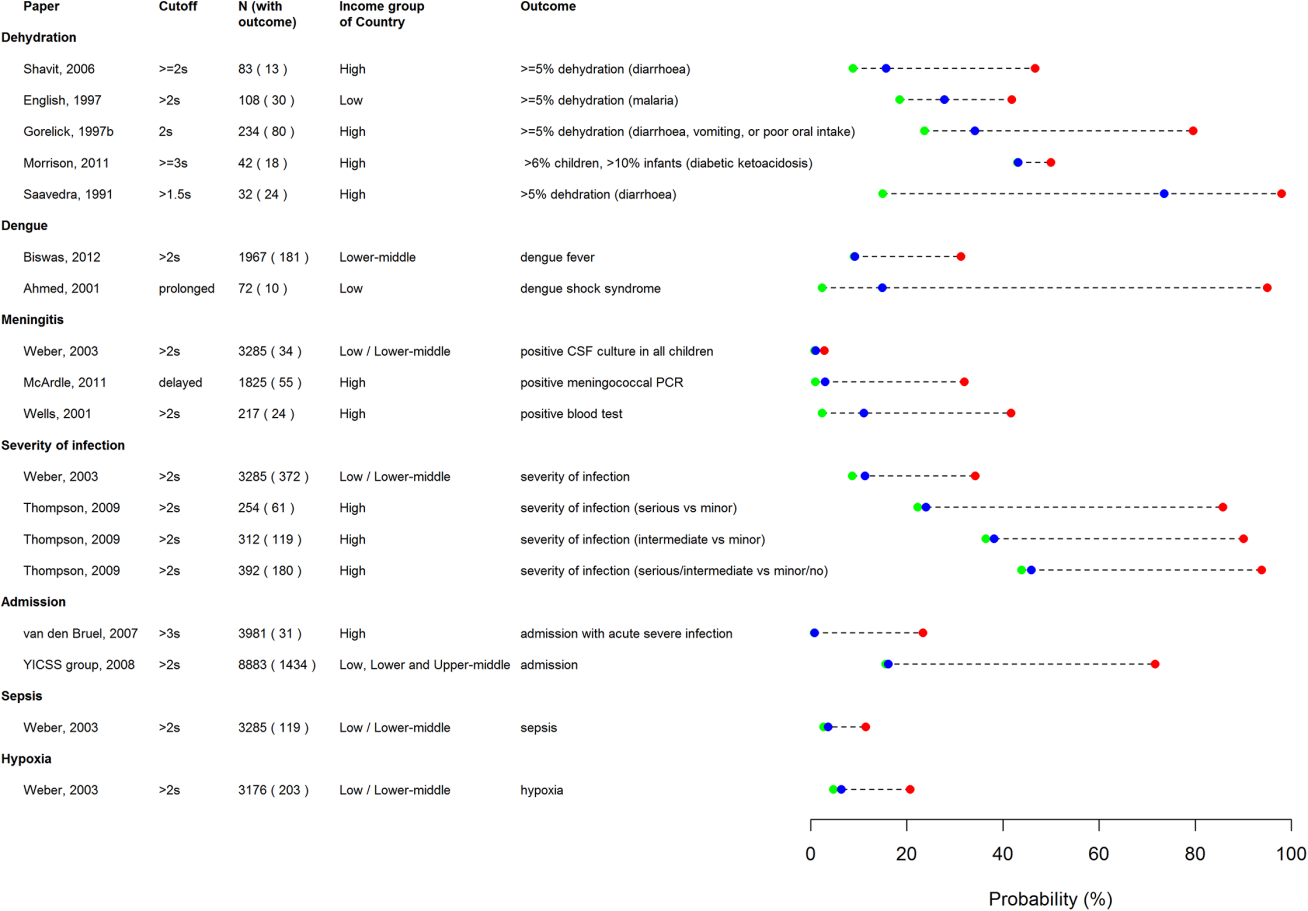
Pre/post test probability plot of prolonged CRT for predicting significant dehydration and severe illness.

We were able to extract sufficient data to reconstruct 2x2 tables for the outcome of ≥ 5% dehydration from all five studies. One study investigated the relationship between CRT and dehydration in children with diabetic ketoacidosis.[26] However, given that dehydration as a result of diabetic ketoacidosis has a markedly different pathophysiology from other causes of dehydration (e.g. infectious gastroenteritis), we did not include this study in the meta-analysis but it is presented in the dumbbell plot. The three studies reporting data for children attending Emergency Departments with gastrointestinal symptoms[25, 27, 28] all showed high specificity (range 88–94%). A hierarchical summary ROC curve for this data is shown in S1 Fig. The dumbbell plot in Fig 5 shows that prolonged CRT markedly increases the probability of a child having ≥ 5% dehydration, except in the case of diabetic ketoacidosis, where the utility of CRT is greatly reduced. Positive likelihood ratios ranged from 1.3 to 16.9, with negative likelihood ratios ranging from 0.06 to 0.99 (S5 Table).

The high heterogeneity of the data, as evidenced by the large confidence and prediction regions and the large distance of the individual study results from the summary ROC curve, means that reporting of the position of the summary point is not statistically relevant.

### Diagnostic accuracy of CRT for other serious illnesses

We identified 11 studies with data on the accuracy of CRT for eight other clinical outcomes: dengue, meningitis, admission, sepsis, urinary tract infection, pneumonia, hypoxia, and severity of illness. Leonard and Beattie[29] reported no significant association between CRT and meningococcal disease or other significant bacterial illness but did not provide specific data (and contact with the authors failed to yield additional data). Three further studies did not report 2x2 tables for any outcomes;[19, 30, 31] data were obtained after contact with two study authors,[19, 31] but attempts to obtain results from the third study[30] were unsuccessful, although odds ratios are reported for all outcomes. One study[32] was excluded from the pre/post test probability plot as the case-control design precluded calculation of true prevalence. The results obtained are summarized in S6 Table.

Prolonged CRT shows consistently high specificity for prediction of dengue, meningitis, admission, sepsis, hypoxia, and severity of illness. No sensitivity or specificity values could be calculated for the outcomes of urinary tract infection or pneumonia, but reported odds ratios of between 2 and 5 are similar to those for other outcomes. In two studies of dengue, prolonged CRT showed variable sensitivity, which is likely to be explained by the large differences in the sample size and study design. Prolonged CRT showed low sensitivity for meningitis in one study carried out in low and lower-middle income settings, but moderate sensitivity in two studies carried out in high income settings. The sensitivity of CRT was relatively low for prediction of admission, sepsis, hypoxia, and severity of illness.

The pre/post test probability plots (Fig 5) for the eight studies for which prevalence and likelihood ratios were available, show that in most cases, prolonged CRT considerably increases the probability of an adverse clinical outcome. However, this is not the case for the study of meningitis in low and lower-middle income countries, although it does appear to hold for studies of meningitis in high-income countries. Few studies show any noticeable reduction in the probability of an adverse outcome following a normal CRT result, even where prevalence is relatively high.

## Discussion

Our results show that CRT is an important “red flag” vital sign for identifying children with serious illness: finding an abnormal CRT increases the likelihood of a serious outcome including death and dehydration, but a normal CRT does not make a serious outcome less likely. Therefore, a normal CRT should not be used to rule out serious illness in children.

Our review identified 24 studies on over 53,000 children across a variety of outcomes and settings including primary and secondary care, low- and high-income countries, and emergency services. A prolonged CRT consistently performed well as a red flag in all these different situations, but the most robust evidence is found in low or middle income settings. Considering it is a clinical feature that does not require sophisticated equipment and can be measured in children of all ages in all circumstances, the measurement of CRT should be part of the standard evaluation of all acutely ill children worldwide.

The most convincing data were found for predicting death, indicating that a child with a prolonged CRT has a 4-fold greater risk of dying compared to a child with normal CRT. This strongly reinforces the recommendations in the World Health Organization’s Integrated Management of Childhood Illness (IMCI) guidelines that an abnormal CRT indicates shock and should be managed accordingly, and suggests that measurement of CRT should become part of routine IMCI protocols.[3] However, all of these studies were carried out in high mortality settings (lower income countries or high acuity settings in high income countries), and are unlikely to be generalizable to settings with low mortality rates.

Although heterogeneity precluded drawing firm conclusions from meta-analysis, abnormal CRT was shown to markedly increase the post-test probability of significant dehydration, with the exception of children presenting with diabetic ketoacidosis. The majority of these studies were performed in high-income settings, and reaffirm the importance of routinely measuring CRT in children with suspected dehydration, a common reason why parents bring children to healthcare professionals.

CRT specificity may vary for certain diseases. For example, it was less useful for meningitis than for sepsis, dengue, hypoxia, or need for hospital admission for serious infection. This may be because of the rapid evolving illness in meningitis leaving a narrow window for delivering life-saving treatments, or due to the infrequent presence of shock in these situations, as reduced peripheral perfusion secondary to shock is likely to be a common cause of prolonged CRT. Prolonged CRT clearly has a diagnostic role in assessing children, but we found a somewhat lower positive likelihood ratio (4.5 for outcome of mortality) compared to other markers of serious illness in children in high income settings (such as tachypnea, body temperature ≥ 40°C, or meningeal irritation[12, 13]) where positive likelihood ratios typically range between 5 and 10. Although likelihood ratios of 5 are generally considered to only moderately change the probability of disease, this may be all that can be expected from a clinical feature.[33] Moreover, the inconsistent and non-standardized measurement of CRT in clinical practice may also reduce the potential utility of this vital sign.[34, 35]

To our knowledge, this is the first systematic review of the diagnostic accuracy of CRT in children worldwide. We used a comprehensive search strategy that identified all available studies. However, studies often evaluate CRT together with many other clinical features, and thus terms referring to CRT or peripheral perfusion may not have picked up all available studies. We therefore supplemented our literature searches by reference tracking and expert consultation. We were unable to assess our results for publication bias, as there were fewer than 10 studies for any given outcome.

We found substantial heterogeneity particularly between studies reporting CRT for predicting dehydration. The statistical heterogeneity cannot be fully explained by clinical factors alone, but may be partly due to differences in measurement technique, which were incompletely reported. Nevertheless, the results of the individual studies suggest that while CRT could be an important red flag for dehydration, more evidence from better specified and appropriately powered studies are needed to obtain a precise estimate of CRT’s diagnostic value.

Poor reporting was a significant issue across included studies. In particular, we found inconsistent or incomplete reporting on how CRT was measured. The results of our previous review indicate that measurement methods such as the choice of body site, pressing time or ambient temperature impact the test’s validity and reliability.[6] The paucity of reporting of these factors suggests that the estimates of diagnostic accuracy we report may be more conservative than if the studies had used more consistent and valid measurement methods. However, where reported, included studies used the finger as the measurement site, and use cut-offs of 2 or 3 seconds to define prolonged CRT. These are consistent with the findings of our previous review, which recommends that CRT should be measured on the finger, and found that normal CRT in healthy children at this site is 2 seconds or less. Standardization of CRT measurement is essential for future diagnostic accuracy studies.

There was also poor reporting of whether all included children received measurements of both CRT and the clinical outcome, which was typically due to retrospective or observational study designs, leading to missing data. This incomplete reporting introduces both risk of selection bias and may limit the representativeness of the findings as children who were particularly unwell may have been excluded in the studies. We were unable to assess the diagnostic value of CRT in detecting serious illness at different age groups, and the inclusion of wide age ranges in some studies may have obscured potential age-related differences. We also found very limited data from primary care settings where access to diagnostic investigations may be limited and clinical signs such as CRT play a vital role in clinical assessment.

For clinicians, our findings emphasize the diagnostic value of CRT; highlighting that CRT is generally highly specific, but has variable and often low sensitivity–hence better for ruling in than ruling out. Since CRT has limited reliability, repeating measurement is important before getting alarmed. Clinical guidelines need to emphasize their rationale for including CRT, including the method for measurement (making it clearer where and for how long to press, and use of a stopwatch, or equivalent, to measure), and the interpretation of CRT values (avoiding terms like prolonged or sluggish, and instead provide whole second cut off values).

A number of important issues remain unresolved. First, we found only one study in primary care, and no studies that looked at the outcomes of dehydration in this setting. Yet, the numbers presenting with acute gastroenteritis and dehydration in these settings worldwide are considerable. Second, using more consistent and standardized methods of measuring CRT to determine its clinical utility across a wide range of diseases could aid its interpretation and use. Third, studies are needed to determine the added diagnostic value of CRT or its independent diagnostic value over and above other vital signs and clinical features. Finally, there are opportunities to explore the use of new technologies to improve the measurement and detection of CRT, and determine whether they perform better when compared with heart rate or other non-invasive measures of cardiovascular status such as blood pressure.

## Conclusions

A capillary refill time of 3 seconds or more is an important warning sign for serious illness and risk of death in children, and can easily be used in a wide range of settings and with only minimal training. Normal CRT in children does not make a serious outcome less likely, and should not be used to rule out serious illness. Standardization of CRT measurement would likely increase the diagnostic utility of this test.

## Supporting Information

S1 Fig Hierarchical summary ROC curve showing diagnostic accuracy of CRT for predicting significant dehydration. (PDF)

S1 Table Quality assessment criteria. (PDF)

S2 Table Characteristics of included studies. (PDF)

S3 Table Quality assessment of included papers. (PDF)

S4 Table Extracted data on diagnostic accuracy of CRT for predicting mortality in children. (PDF)

S5 Table Extracted data on diagnostic accuracy of CRT for ≥5% dehydration. (PDF)

S6 Table Diagnostic accuracy of CRT in predicting serious illness and admission to hospital. (PDF)

S1 Text Systematic review protocol. (PDF)

S2 Text Search strategy for diagnostic accuracy studies. (PDF)

S3 Text Completed PRISMA checklist. (PDF)
