# Supplementary material for: The Diagnostic Value of Capillary Refill Time for Detecting Serious Illness in Children: A Systematic Review and Meta-Analysis
Source: PLoS One. 2015 Sep 16;10(9):e0138155. doi: 10.1371/journal.pone.0138155 (PMC4573516; doi:10.1371/journal.pone.0138155)

S1 Fig: Hierarchical summary ROC curve showing diagnostic accuracy of CRT for predicting significant dehydration

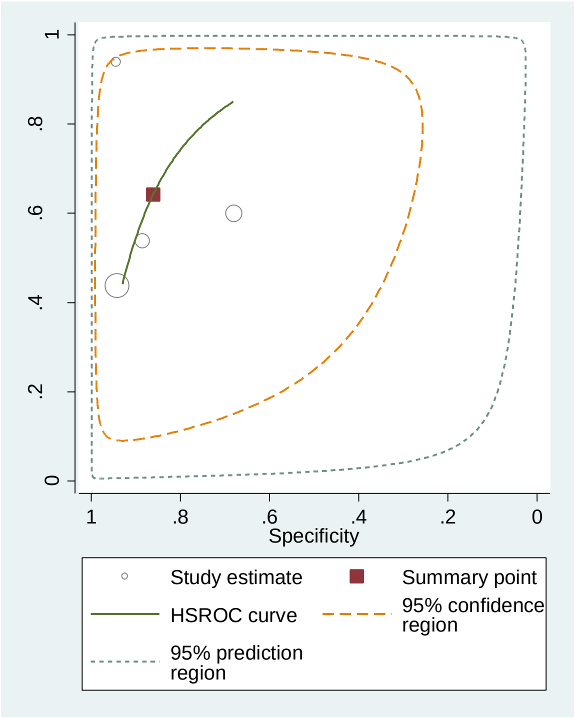

Supplement: S1 Fig — (PDF) [file pone.0138155.s001.pdf]
