# Supplementary material for: The Diagnostic Value of Capillary Refill Time for Detecting Serious Illness in Children: A Systematic Review and Meta-Analysis
Source: PLoS One. 2015 Sep 16;10(9):e0138155. doi: 10.1371/journal.pone.0138155 (PMC4573516; doi:10.1371/journal.pone.0138155)
Supplement: S1 Table — (PDF) [file pone.0138155.s002.pdf]

S1 Table: Quality assessment criteria

Patient selection	Bias: suitable sampling method used
	Bias: appropriate exclusion criteria
	Applicability: appropriate inclusion criteria
Index test (CRT)	Bias: blinded to result of reference standard
	Bias: time measured OR pre-specified threshold used
	Applicability: site and time measurement method defined
Reference standard (clinical outcome)	Bias: blinded to index test result
	Bias: independent of result of index test
	Applicability: objective measurement
Timing and flow	Bias: contemporaneous measurement of index test and reference standard
	Bias: all children had index test performed
	Bias: all children had reference standard assessed
