# Supplementary material for: The Diagnostic Value of Capillary Refill Time for Detecting Serious Illness in Children: A Systematic Review and Meta-Analysis
Source: PLoS One. 2015 Sep 16;10(9):e0138155. doi: 10.1371/journal.pone.0138155 (PMC4573516; doi:10.1371/journal.pone.0138155)
Supplement: S2 Table — (PDF) [file pone.0138155.s003.pdf]

S2 Table: Characteristics of included studies

Study	Setting	Population	Age range	Sample size	Type / Recruitment	CRT measurement method and cutoff(s) used	Main outcomes
**Ahmed, 2001[1]**	Urban teaching hospital, Bangladesh	Children admitted to paediatric ward with dengue	2.5-12 years	72 (5 died)	Observational / consecutive	"Prolonged" cutoff	Death, dengue shock syndrome
**Biswas, 2012[2]**	Community and health centre, Nicaragua	Children enrolled in cohort brought to health centre at first sign of illness or fever	2-14 years	4464 (1974 episodes of potential dengue fever), 1967 with CRT	Cohort / pre-defined cohort	>2 seconds cutoff	Laboratory confirmation of dengue virus infection
**Carcillo, 2009[3]**	Speciality transport teams, USA	Infants and children referred from community hospitals for transport to paediatric centres	0-18 years	4766 (283 died)	Observational / consecutive	>3 seconds cutoff	Death
**Clifton, 2012[4]**	Tertiary referral hospital, Moshi, Tanzania	Children admitted as inpatients with history of fever in last 48 hours, axillary temperature ≥38.5C or rectal temperature ≥38C	2 months-13 years	466 (34 died)	Observational / consecutive	>3 seconds cutoff	Death
**Craig, 2010[5]**	Paediatric emergency department, Westmead, Australia	Children presenting to emergency department with febrile illness	0-5 years	15781 illnesses in 12807 children (1140 with serious bacterial infection)	Observational / consecutive	>2 seconds, 2-3 seconds, >3 seconds	Urinary tract infection, bacteraemia, pneumonia
**English, 1997[6]**	Rural district hospital, Kenya	Children with sole diagnosis of falciparum malaria, who were prostrated, in coma, or had respiratory distress	>1 month	108 (29 with >=5% dehydration)	Observational / consecutive	Finger pulp, >2 seconds cutoff	>=5% fluid deficit
**Evans, 2006[7]**	Tertiary teaching hospital, Ghana	Paediatric patients screening positive for asexual P falciparum parasitaemia, with Haemoglobin<5g/dl, lactate>5mmol/L, or BCS (coma score)<3	3 months to >60 months	2446 (172 died), 2342 with CRT	Observational / Consecutive	3 seconds pressure on finger, >2 seconds cutoff	Death
**Gorelick, 1997a[8]**	Paediatric ED, USA	Children attending ED with vomiting, diarrhoea or poor oral intake	1 month -5 years	186 (116 admitted)	Observational / convenience	Fingertip, measured with stopwatch, mean of 3 measurements measured by ED nurses or researchers, >2 seconds cutoff	>=5% and >=10% fluid deficit
**Gorelick, 1997b[9]**	Paediatric ED, USA	Children attending ED with vomiting, diarrhoea or poor oral intake	1 month -5 years	234 (174 admitted)	Observational / convenience	5 seconds moderate pressure on finger with hand at level of heart, measured with stopwatch, mean of 3 measurements by ED	>=5% fluid deficit

						nurses, >2 seconds cutoff	
**Kumar, 2003[10]**	Tertiary hospital, India	Patients admitted to hospital	0 to >60 months	1099 (44 died)	Observational / consecutive	>=3 seconds cutoff	Death
**Leonard, 2004[11]**	Paediatric ED, UK	Children with recent onset of illness attending paediatric ED with no trauma	0 to >12 years	4878 (246 admitted)	Observational / consecutive	5 seconds pressure on finger with hand just above level of heart, measured by counting whole seconds by experience paediatric triage nurses	Meningococcal disease, serious bacterial infection other than meningococcal disease, admission
**Maitland, 2006[12]**	Rural district hospital, Kenya	Children older than 3 months admitted to paediatric ward with severe malnutrition	>3 months	920 (176 died)	Observational / consecutive	Fingernail bed, >2 seconds and >3 seconds cutoffs, measured by members of clinical research team	Death
**Mathur, 2007[13]**	Neonatal unit, urban teaching hospital, India	Out-born neonates	Neonates	175 (60 died)	Observational / convenience	>=3 seconds cutoff, most abnormal value in first 12 hours of stay used	Death
**McArdle, 2011[14]**	ED, Ireland	Patients with meningococcal or pneumococcal PCR results	Unclear	1825 (55 with positive PCR)	Case-control / retrospective	"Delayed" cutoff	Positive meningococcal or pneumococcal PCR
**Morrison, 2011[15]**	Tertiary children's hospital, Canada	Children presenting to emergency department, diagnosed with diabetic ketoacidosis	1 month – 16 years	39 (42 episodes of DKA)	Observational / not specified	Not specified	Fluid deficit
**Pamba, 2004[16]**	Rural district hospital, Kenya	Children admitted to paediatric ward or HDU with malaria, malarial anaemia, acute respiratory tract infection, malnutrition, gastroenteritis, anaemia, meningitis, or sepsis	>3 months	4160 (189 died)	Observational / retrospective	3 seconds pressure on finger, measured by counting, >3 seconds cutoff	Death
**Saavedra, 1991[17]**	Tertiary hospital, USA	Admitted to infant ward with history of diarrhoea (32 infants)	1 week – 26 months	32 (24 with deficit >50mL/kg)	Observational / not specified	Light pressure to nail bed, >1.5 seconds and >3 seconds cutoffs	>5%, 5-10%, and >10% fluid deficit
**Shavit, 2006[18]**	Paediatric ED, Canada	Children with a history of diarrhoea, judged to have some degree of dehydration	1 month – 5 years	83 with diarrhoea (1 admitted)	Observational / convenience	"Standard clinical techniques" with >=2 seconds cutoff by staff physicians	>=5% fluid deficit
**Thompson, 2009[19]**	Paediatric assessment unit, UK	Children whose parents, referring clinician, or triage nurse suspected acute infection	3 months – 16 years	700 (383 admitted), 392 with CRT (180 with serious infection)	Observational / not specified	>2 seconds cutoff	Severity of infection (serious, intermediate or minor)
**van den Bruel, 2007[20]**	Primary care, Flanders, Belgium	Children consulting GP, paediatrician, or ED, not referred by another physician, with acute illness for a maximum of 5 days	0-16 years	3981 (31 with serious infection)	Observational / consecutive	>3 seconds cutoff	Admission to hospital with acute serious infection
**Verbakel, 2014[21]**	Hospital, UK	Children admitted to hospital after ED attendance or referral by GP	0-16 years	857 (50 with meningitis or sepsis)	Case-control / consecutive (half of	>=3 seconds cutoff	Positive sterile site culture with a final discharge diagnosis of meningitis,

					controls selected at random for analysis)		bacteraemia, or sepsis
**Weber, 2003[22]**	Tertiary hospitals, Ethiopia, the Gambia, Papua New Guinea, and the Philippines	Infants with a wide spectrum of illness severity	0-59 days	3285 (197 died)	Observational / consecutive	>2 seconds cutoff	Death, sepsis (growth of known pathogen in blood), meningitis (positive CSF culture), hypoxaemia <90%, severity of outcome judged by clinicians on 3-point scale ranging from no abnormality to severe hypoxaemia, bacteraemia, or meningitis
**Wells, 2001[23]**	Paediatric ED, UK	All infants and children presenting to ED with non-blanching rash	0-15 years	218 (1 died), 217 with CRT	Observational / consecutive	>=2 seconds cutoff	Meningococcal infection defined by positive blood test
**YICSS group, 2008[24]**	Urban teaching hospitals, Bangladesh, Bolivia, Ghana, India, South Africa, and Pakistan	Infants brought to hospital or outpatient clinic for acute illness	0-60 days	8889 (68 died), 8883 with CRT	Observational / consecutive	"Prolonged" cutoff	Severe illness defined by a need for hospital treatment as judged by a clinician

Abbreviations used: ED – Emergency Department, GP – General practitioner, HDU – high dependency unit

Reference List

1. Ahmed F, Mahmood C, Sharma J, Hoque S, Zaman R, et al. (2001) Dengue and dengue haemorrhagic fever in children during the 2000 outbreak in Chittagong, Bangladesh. Dengue Bulletin 25: 33-39.
2. Biswas HH, Ortega O, Gordon A, Standish K, Balmaseda A, et al. (2012) Early clinical features of dengue virus infection in nicaraguan children: a longitudinal analysis. PLoS Neglected Tropical Diseases [electronic resource] 6: e1562.
3. Carcillo JA, Kuch BA, Han YY, Day S, Greenwald BM, et al. (2009) Mortality and functional morbidity after use of PALS/APLS by community physicians. Pediatrics 124: 500-508.
4. Clifton DC, Ramadhani HO, Msuya LJ, Njau BN, Kinabo GD, et al. (2012) Predicting mortality for paediatric inpatients where malaria is uncommon. Archives of disease in childhood 97: 889-894.
5. Craig JC, Williams GJ, Jones M, Codarini M, Macaskill P, et al. (2010) The accuracy of clinical symptoms and signs for the diagnosis of serious bacterial infection in young febrile children: prospective cohort study of 15 781 febrile illnesses. BMJ 340: c1594.
6. English M, Waruiru C, Mwakesi R, Marsh K (1997) Signs of dehydration in severe childhood malaria. Tropical doctor 27: 235-236.
7. Evans JA, May J, Ansong D, Antwi S, Asafo-Adjei E, et al. (2006) Capillary refill time as an independent prognostic indicator in severe and complicated malaria. The Journal of pediatrics 149: 676-681.
8. Gorelick MH, Shaw KN, Murphy KO (1997) Validity and reliability of clinical signs in the diagnosis of dehydration in children. Pediatrics 99: E6.
9. Gorelick MH, Shaw KN, Murphy KO, Baker MD (1997) Effect of fever on capillary refill time. Pediatric emergency care 13: 305-307.
10. Kumar N, Thomas N, Singhal D, Puliyel JM, Sreenivas V (2003) Triage score for severity of illness. Indian pediatrics 40: 204-210.
11. Leonard PA, Beattie TF (2004) Is measurement of capillary refill time useful as part of the initial assessment of children? European Journal of Emergency Medicine 11: 158-163.

12. Maitland K, Berkley JA, Shebbe M, Peshu N, English M, et al. (2006) Children with severe malnutrition: can those at highest risk of death be identified with the WHO protocol? PLoS medicine 3: e500.
13. Mathur NB, Arora D (2007) Role of TOPS (a simplified assessment of neonatal acute physiology) in predicting mortality in transported neonates. Acta Paediatr 96: 172-175.
14. McArdle S, O'Sullivan R, Walsh S (2011) Utility of PCR testing for invasive meningococcal and pneumococcal disease in a paediatric emergency care setting. Academic Emergency Medicine Conference: 2011 Annual Meeting of the Society for Academic Emergency Medicine, SAEM Boston, MA United States. Conference Start: 20110601 Conference End: 20110605. Conference Publication:: S178.
15. Morrison G, Sottosanti M, Singh R, Sharma A, Fraser D, et al. (2011) Quantifying dehydration in children with diabetic ketoacidosis (DKA): Does it matter? Pediatr Crit Care Me Conference: 6th World Congress on Pediatric Critical Care: One World Sharing Knowledge Sydney, NSW Australia. Conference Start: 20110313 Conference End: 20110317. Conference Publication:: A122-A123.
16. Pamba A, Maitland K (2004) Capillary refill: prognostic value in Kenyan children. Archives of disease in childhood 89: 950-955.
17. Saavedra JM, Harris GD, Li S, Finberg L (1991) Capillary refilling (skin turgor) in the assessment of dehydration. Am J Dis Child 145: 296-298.
18. Shavit I, Brant R, Nijssen-Jordan C, Galbraith R, Johnson DW (2006) A novel imaging technique to measure capillary-refill time: improving diagnostic accuracy for dehydration in young children with gastroenteritis. Pediatrics 118: 2402-2408.
19. Thompson M, Coad N, Harnden A, Mayon-White R, Perera R, et al. (2009) How well do vital signs identify children with serious infections in paediatric emergency care? Archives of disease in childhood 94: 888-893.
20. Van den Bruel A, Aertgeerts B, Bruyninckx R, Aerts M, Buntinx F (2007) Signs and symptoms for diagnosis of serious infections in children: a prospective study in primary care. The British journal of general practice : the journal of the Royal College of General Practitioners 57: 538-546.
21. Verbakel JY, MacFaul R, Aertgeerts B, Buntinx F, Thompson M (2014) Sepsis and meningitis in hospitalized children: performance of clinical signs and their prediction rules in a case-control study. Pediatric emergency care 30: 373-380.
22. Weber MW, Carlin JB, Gatchalian S, Lehmann D, Muhe L, et al. (2003) Predictors of neonatal sepsis in developing countries. Pediatr Infect Dis J 22: 711-717.
23. Wells LC, Smith JC, Weston VC, Collier J, Rutter N (2001) The child with a non-blanching rash: how likely is meningococcal disease? Archives of disease in childhood 85: 218-222.
24. Young Infants Clinical Signs Study G (2008) Clinical signs that predict severe illness in children under age 2 months: a multicentre study. Lancet 371: 135-142.
