# Supplementary material for: The Diagnostic Value of Capillary Refill Time for Detecting Serious Illness in Children: A Systematic Review and Meta-Analysis
Source: PLoS One. 2015 Sep 16;10(9):e0138155. doi: 10.1371/journal.pone.0138155 (PMC4573516; doi:10.1371/journal.pone.0138155)
Supplement: S3 Table — (PDF) [file pone.0138155.s004.pdf]

S3 Table: Quality assessment of included papers

	Patient selection			Index test (CRT)			Reference standard (clinical outcome)			Timing and flow		
	sampling method	exclusion criteria	inclusion criteria	CRT blinding	time measured	defined site and time	reference standard blinding	reference standard objective	reference standard independent	contemporaneous	all children had CRT	all children had reference standard
Ahmed, 2001[1]	✓	?	✓	?	X	X	?	?	?	?	✓	✓
Biswas, 2012[2]	✓	✓	✓	?	✓	?	?	✓	✓	X	?	?
Carcillo, 2009[3]	✓	?	✓	?	✓	?	?	?	?	?	✓*	?
Clifton, 2012[4]	✓	✓	✓	✓	✓	?	?	✓	✓	X	✓	✓
Craig, 2010[5]	✓	✓	✓	✓	✓	X	?	✓	✓	X	✓	✓
English, 1997[6]	✓	✓	✓	✓	✓	✓	?	✓	✓	X	X	X
Evans, 2006[7]	✓	✓	✓	✓	✓	✓	?	✓	✓	X	✓*	✓
Gorelick, 1997a[8]	✓	✓	✓	✓	✓	✓	?	✓	✓	X	✓	X
Gorelick, 1997b[9]	✓	✓	✓	?	✓	✓	?	✓	✓	✓	✓	X
Kumar, 2003[10]	✓	✓	✓	✓	✓	X	?	✓	✓	X	✓	✓
Leonard, 2004[11]	✓	✓	✓	✓	✓	✓	?	✓	?	X	X	?
Maitland, 2006[12]	✓	✓	✓	✓	✓	?	?	✓	✓	X	✓	✓
Mathur, 2007[13]	✓	✓	✓	✓	✓	X	?	✓	✓	X	?	?
McArdle, 2011[14]	✓	?	✓	✓	X	X	?	✓	✓	?	?	✓
Morrison, 2011[15]	✓	✓	✓	✓	?	X	?	✓	✓	X	?	?
Pamba, 2004[16]	✓	✓	✓	✓	✓	✓	?	✓	✓	X	✓*	✓
Saavedra, 1991[17]	?	✓	✓	✓	?	?	?	✓	✓	X	✓	✓
Shavit, 2006[18]	✓	✓	✓	✓	✓	✓	X	✓	✓	✓	✓	X
Thompson, 2009[19]	✓	✓	✓	?	✓	X	?	?	?	X	X	✓
van den Bruel, 2007[20]	✓	✓	✓	✓	✓	?	?	✓	✓	X	✓	✓
Verbakel, 2014[21]	X	✓	✓	✓	✓	X	X	✓	?	X	X	✓
Weber, 2003[22]	✓	✓	✓	?	✓	X	?	✓	✓	?	?	?
Wells, 2001[23]	✓	✓	✓	?	✓	X	?	✓	✓	?	✓*	✓

YICSS group, 2008[24]	✓	✓	✓	?	?	X	?	?	?	X	?	✓

? used for "unclear"

** some studies had >95% children with CRT measurements, and were judged to be at low risk of bias due to missing CRT data*

Reference List

1. Ahmed F, Mahmood C, Sharma J, Hoque S, Zaman R, et al. (2001) Dengue and dengue haemorrhagic fever in children during the 2000 outbreak in Chittagong, Bangladesh. Dengue Bulletin 25: 33-39.
2. Biswas HH, Ortega O, Gordon A, Standish K, Balmaseda A, et al. (2012) Early clinical features of dengue virus infection in nicaraguan children: a longitudinal analysis. PLoS Neglected Tropical Diseases [electronic resource] 6: e1562.
3. Carcillo JA, Kuch BA, Han YY, Day S, Greenwald BM, et al. (2009) Mortality and functional morbidity after use of PALS/APLS by community physicians. Pediatrics 124: 500-508.
4. Clifton DC, Ramadhani HO, Msuya LJ, Njau BN, Kinabo GD, et al. (2012) Predicting mortality for paediatric inpatients where malaria is uncommon. Archives of disease in childhood 97: 889-894.
5. Craig JC, Williams GJ, Jones M, Codarini M, Macaskill P, et al. (2010) The accuracy of clinical symptoms and signs for the diagnosis of serious bacterial infection in young febrile children: prospective cohort study of 15 781 febrile illnesses. BMJ 340: c1594.
6. English M, Waruiru C, Mwakesi R, Marsh K (1997) Signs of dehydration in severe childhood malaria. Tropical doctor 27: 235-236.
7. Evans JA, May J, Ansong D, Antwi S, Asafo-Adjei E, et al. (2006) Capillary refill time as an independent prognostic indicator in severe and complicated malaria. The Journal of pediatrics 149: 676-681.
8. Gorelick MH, Shaw KN, Murphy KO (1997) Validity and reliability of clinical signs in the diagnosis of dehydration in children. Pediatrics 99: E6.
9. Gorelick MH, Shaw KN, Murphy KO, Baker MD (1997) Effect of fever on capillary refill time. Pediatric emergency care 13: 305-307.
10. Kumar N, Thomas N, Singhal D, Puliyel JM, Sreenivas V (2003) Triage score for severity of illness. Indian pediatrics 40: 204-210.
11. Leonard PA, Beattie TF (2004) Is measurement of capillary refill time useful as part of the initial assessment of children? European Journal of Emergency Medicine 11: 158-163.
12. Maitland K, Berkley JA, Shebbe M, Peshu N, English M, et al. (2006) Children with severe malnutrition: can those at highest risk of death be identified with the WHO protocol? PLoS medicine 3: e500.
13. Mathur NB, Arora D (2007) Role of TOPS (a simplified assessment of neonatal acute physiology) in predicting mortality in transported neonates. Acta Paediatr 96: 172-175.
14. McArdle S, O'Sullivan R, Walsh S (2011) Utility of PCR testing for invasive meningococcal and pneumococcal disease in a paediatric emergency care setting. Academic Emergency Medicine Conference: 2011 Annual Meeting of the Society for Academic Emergency Medicine, SAEM Boston, MA United States. Conference Start: 20110601 Conference End: 20110605. Conference Publication:: S178.
15. Morrison G, Sottosanti M, Singh R, Sharma A, Fraser D, et al. (2011) Quantifying dehydration in children with diabetic ketoacidosis (DKA): Does it matter? Pediatr Crit Care Me Conference: 6th World Congress on Pediatric Critical Care: One World Sharing Knowledge Sydney, NSW Australia. Conference Start: 20110313 Conference End: 20110317. Conference Publication:: A122-A123.
16. Pamba A, Maitland K (2004) Capillary refill: prognostic value in Kenyan children. Archives of disease in childhood 89: 950-955.
17. Saavedra JM, Harris GD, Li S, Finberg L (1991) Capillary refilling (skin turgor) in the assessment of dehydration. Am J Dis Child 145: 296-298.
18. Shavit I, Brant R, Nijssen-Jordan C, Galbraith R, Johnson DW (2006) A novel imaging technique to measure capillary-refill time: improving diagnostic accuracy for dehydration in young children with gastroenteritis. Pediatrics 118: 2402-2408.
19. Thompson M, Coad N, Harnden A, Mayon-White R, Perera R, et al. (2009) How well do vital signs identify children with serious infections in paediatric emergency care? Archives of disease in childhood 94: 888-893.
20. Van den Bruel A, Aertgeerts B, Bruyninckx R, Aerts M, Buntinx F (2007) Signs and symptoms for diagnosis of serious infections in children: a prospective study in primary care. The British journal of general practice : the journal of the Royal College of General Practitioners 57: 538-546.

21. Verbakel JY, MacFaul R, Aertgeerts B, Buntinx F, Thompson M (2014) Sepsis and meningitis in hospitalized children: performance of clinical signs and their prediction rules in a case-control study. Pediatric emergency care 30: 373-380.
22. Weber MW, Carlin JB, Gatchalian S, Lehmann D, Muhe L, et al. (2003) Predictors of neonatal sepsis in developing countries. Pediatr Infect Dis J 22: 711-717.
23. Wells LC, Smith JC, Weston VC, Collier J, Rutter N (2001) The child with a non-blanching rash: how likely is meningococcal disease? Archives of disease in childhood 85: 218-222.
24. Young Infants Clinical Signs Study G (2008) Clinical signs that predict severe illness in children under age 2 months: a multicentre study. Lancet 371: 135-142.
