# Supplementary material for: The Diagnostic Value of Capillary Refill Time for Detecting Serious Illness in Children: A Systematic Review and Meta-Analysis
Source: PLoS One. 2015 Sep 16;10(9):e0138155. doi: 10.1371/journal.pone.0138155 (PMC4573516; doi:10.1371/journal.pone.0138155)
Supplement: S4 Table — (PDF) [file pone.0138155.s005.pdf]

S4 Table: Diagnostic accuracy of CRT for predicting mortality in children

Paper	Setting	Population	Sample size (number of deaths)	CRT cutoff	Sensitivity (95% CI)	Specificity (95% CI)	Positive likelihood ratio (95% CI)	Negative likelihood ratio (95% CI)
Ahmed, 2001[1]	Urban teaching hospital, Bangladesh	Children admitted with dengue	72 (5)	"prolonged"	60 (15-95)	91 (82-97)	6.7 (2.4-19.1)	0.44 (0.15-1.29)
Carcillo, 2009[2]	Speciality transport teams, USA	Infants and children referred for transport to paediatric centres	4766 (283)	>3s	62 (56-68)	80 (78-81)	3.0 (2.7-3.4)	0.48 (0.41-0.56)
Clifton, 2012[3]	Tertiary referral hospital, Moshi, Tanzania	Children admitted with history of fever	466 (34)	>3s	18 (7-35)	98 (96-99)	7.6 (2.9-19.7)	0.84 (0.72-0.99)
Evans, 2006[4]	Tertiary teaching hospital, Ghana	Children with malarial parasitaemia, with anamia, lactate>5mmol/L or reduced consciousness	2342 (161)	>2s	28 (21-36)	86 (85-88)	2.1 (1.6-2.7)	0.83 (0.76-0.92)
Kumar, 2003[5]	Tertiary hospital, India	Children admitted to hospital	1099 (44)	>=3s	34 (20-50)	94 (92-95)	5.5 (3.4-8.7)	0.70 (0.57-0.87)
Maitland, 2006[6]	Rural district hospital, Kenya	Children admitted with severe malnutrition	920 (176)	>3s	15 (10-22)	94 (92-95)	2.4 (1.6-3.8)	0.90 (0.85-0.96)
Maitland, 2006[6]	Rural district hospital, Kenya	Children admitted with severe malnutrition	920 (176)	>2s	34 (27-42)	80 (77-83)	1.7 (1.3-2.2)	0.82 (0.73-0.92)
Mathur, 2007[7]	Neonatal unit, urban teaching hospital, India	Out-born neonates	175 (60)	>=3s	52 (38-65)	97 (91-99)	14.9 (5.5-40.1)	0.5 (0.38-0.65)
Pamba, 2004[8]	Rural district hospital, Kenya	Children admitted with malaria, malarial anaemia, acute respiratory tract infection, malnutrition, gastroenteritis, anaemia, meningitis, or sepsis	4160 (189)	>3s	23 (17-30)	92 (92-93)	3.1 (2.3-4.1)	0.83 (0.77-0.90)
Weber, 2003[9]	Tertiary hospitals, Ethiopia, the Gambia, Papua New Guinea, and the Philippines	Infants with a wide ranges of illness severity	3285 (197)	>2s	51 (44-58)	92 (91-93)	6.3 (5.3-7.6)	0.54 (0.46-0.62)
**Results for subgroups reporting diagnotic accuracy after specific diagnoses**								
Pamba, 2004[8]	Rural district hospital, Kenya	Children admitted with malarial anaemia	424 (6)	>3s	33 (4-78)	83 (80-87)	2.0 (0.6-6.5)	0.80 (0.45-1.40)
Pamba, 2004[8]	Rural district hospital, Kenya	Children admitted with acute respiratory infection	862 (19)	>3s	5 (0-26)	96 (95-98)	1.5 (0.2-10.3)	0.98 (0.88-1.09)
Pamba, 2004[8]	Rural district hospital, Kenya	Children admitted with malaria	1654 (45)	>3s	27 (15-42)	94 (93-95)	4.8 (2.8-8.1)	0.78 (0.65-0.93)

Pamba, 2004[8]	Rural district hospital, Kenya	Children admitted with gastroenteritis	620 (18)	>3s	22 (6-48)	93 (90-95)	3.1 (1.3-7.7)	0.84 (0.65-1.07)
Pamba, 2004[8]	Rural district hospital, Kenya	Children admitted with anaemia	147 (6)	>3s	67 (22-96)	75 (67-82)	2.7 (1.4-5.1)	0.44 (0.14-1.38)
Pamba, 2004[8]	Rural district hospital, Kenya	Children admitted with meningitis	59 (6)	>3s	7 (0-50)	99 (92-100)	7.7 (0.2-359)	0.94 (0.76-1.15)
Pamba, 2004[8]	Rural district hospital, Kenya	Children admitted with malnutrition	373 (81)	>3s	23 (15-34)	88 (84-92)	2.0 (1.2-3.3)	0.87 (0.76-0.98)
Pamba, 2004[8]	Rural district hospital, Kenya	Children admitted with sepsis	21 (8)	>3s	25 (3-65)	85 (55-98)	1.6 (0.3-9.4)	0.89 (0.56-1.41)

Reference List

1. Ahmed F, Mahmood C, Sharma J, Hoque S, Zaman R, et al. (2001) Dengue and dengue haemorrhagic fever in children during the 2000 outbreak in Chittagong, Bangladesh. Dengue Bulletin 25: 33-39.
2. Carcillo JA, Kuch BA, Han YY, Day S, Greenwald BM, et al. (2009) Mortality and functional morbidity after use of PALS/APLS by community physicians. Pediatrics 124: 500-508.
3. Clifton DC, Ramadhani HO, Msuya LJ, Njau BN, Kinabo GD, et al. (2012) Predicting mortality for paediatric inpatients where malaria is uncommon. Archives of disease in childhood 97: 889-894.
4. Evans JA, May J, Ansong D, Antwi S, Asafo-Adjei E, et al. (2006) Capillary refill time as an independent prognostic indicator in severe and complicated malaria. The Journal of pediatrics 149: 676-681.
5. Kumar N, Thomas N, Singhal D, Puliyel JM, Sreenivas V (2003) Triage score for severity of illness. Indian pediatrics 40: 204-210.
6. Maitland K, Berkley JA, Shebbe M, Peshu N, English M, et al. (2006) Children with severe malnutrition: can those at highest risk of death be identified with the WHO protocol? PLoS medicine 3: e500.
7. Mathur NB, Arora D (2007) Role of TOPS (a simplified assessment of neonatal acute physiology) in predicting mortality in transported neonates. Acta Paediatr 96: 172-175.
8. Pamba A, Maitland K (2004) Capillary refill: prognostic value in Kenyan children. Archives of disease in childhood 89: 950-955.
9. Weber MW, Carlin JB, Gatchalian S, Lehmann D, Muhe L, et al. (2003) Predictors of neonatal sepsis in developing countries. Pediatr Infect Dis J 22: 711-717.
