# Supplementary material for: The Diagnostic Value of Capillary Refill Time for Detecting Serious Illness in Children: A Systematic Review and Meta-Analysis
Source: PLoS One. 2015 Sep 16;10(9):e0138155. doi: 10.1371/journal.pone.0138155 (PMC4573516; doi:10.1371/journal.pone.0138155)
Supplement: S5 Table — (PDF) [file pone.0138155.s006.pdf]

S5 Table: Diagnostic accuracy of CRT for ≥5% dehydration

Paper	Setting	Population	Sample size (number with ≥ 5% dehydration)	CRT cutoff	Sensitivity (95% CI)	Specificity (95% CI)	Positive likelihood ratio (95% CI)	Negative likelihood ratio (95% CI)
English, 1997*[1]	Rural district hospital, Kenya	Children with sole diagnosis of falciparum malaria, who were prostrated, in coma, or had respiratory distress	108 (30)	>2s	60 (41-77)	68 (56-78)	1.9 (1.2-2.9)	0.59 (0.37-0.94)
Gorelick, 1997b[2]	Paediatric ED, USA	Children attending with vomiting, diarrhoea or poor oral intake	234 (80)	>2s	44 (33-55)	94 (89-97)	7.5 (3.8-14.8)	0.60 (0.49-0.73)
Morrison, 2011^[3]	Tertiary children's hospital, Canada	Children presenting to ED, diagnosed with diabetic ketoacidosis	42	>=3s	0 (0-22)	100 (80-100)	1.3 (0.0-63.4)	0.99 (0.91-1.09)
Saavedra, 1991+[4]	Tertiary hospital, USA	Admitted to infant ward with history of diarrhoea	32 (24)	>1.5s	94 (77-100)	94 (59-100)	16.9 (1.1-250.6)	0.06 (0.01-0.30)
Shavit, 2006[5]	Paediatric ED, Canada	Children with history of diarrhoea, judged to have some degree of dehydration	83 (13)	>=2s	54 (25-81)	88 (75-93)	4.5 (2-10.4)	0.52 (0.29-0.95)

* values reported in English, 1997 are inconsistent; this 2x2 table represents a probable estimation of the "true" data, as communication with the authors confirmed that the original data were no longer available. However, it was possible to obtain a valid 2x2 table after a small correction to the reported numbers (increasing the number of subjects with each outcome by one), and sensitivity analysis showed that potential errors in the correction had minimal effect on the final result.

^not included in meta-analysis as all children had diabetic ketoacidosis. Dehydration defined as >6% in children and >10% in infants

+ also reported a correlation coefficient of 0.843 (p<0.005) for the comparison of measured CRT against fluid deficit

Reference List

1. English M, Waruiru C, Mwakesi R, Marsh K (1997) Signs of dehydration in severe childhood malaria. Tropical doctor 27: 235-236.
2. Gorelick MH, Shaw KN, Murphy KO, Baker MD (1997) Effect of fever on capillary refill time. Pediatric emergency care 13: 305-307.
3. Morrison G, Sottosanti M, Singh R, Sharma A, Fraser D, et al. (2011) Quantifying dehydration in children with diabetic ketoacidosis (DKA): Does it matter? Pediatr Crit Care Me Conference: 6th World Congress on Pediatric Critical Care: One World Sharing Knowledge Sydney, NSW Australia. Conference Start: 20110313 Conference End: 20110317. Conference Publication:: A122-A123.
4. Saavedra JM, Harris GD, Li S, Finberg L (1991) Capillary refilling (skin turgor) in the assessment of dehydration. Am J Dis Child 145: 296-298.
5. Shavit I, Brant R, Nijssen-Jordan C, Galbraith R, Johnson DW (2006) A novel imaging technique to measure capillary-refill time: improving diagnostic accuracy for dehydration in young children with gastroenteritis. Pediatrics 118: 2402-2408.
