# Supplementary material for: The Diagnostic Value of Capillary Refill Time for Detecting Serious Illness in Children: A Systematic Review and Meta-Analysis
Source: PLoS One. 2015 Sep 16;10(9):e0138155. doi: 10.1371/journal.pone.0138155 (PMC4573516; doi:10.1371/journal.pone.0138155)
Supplement: S6 Table — (PDF) [file pone.0138155.s007.pdf]

S6 Table: Diagnostic accuracy of CRT in predicting serious illness and admission to hospital

Paper	Setting	Population	Sample size (number with outcome)	CRT cutoff	Odds Ratio (95% CI)	Sensitivity (95% CI)	Specificity (95% CI)	Positive likelihood ratio (95% CI)	Negative likelihood ratio (95% CI)
**Prediction of dengue (Biswas) or dengue shock syndrome (Ahmed)**									
Ahmed, 2001[1]	Urban teaching hospital, Bangladesh	Children admitted with dengue	72 (10)	prolonged		86 (53-99)	99 (93-100)	108.8 (6.8-1738)	0.14 (0.03-0.61)
Biswas, 2012[2]	Community and health centre, Nicaragua	Children enrolled in cohort, brought to health centre at first sign of illness or fever	1967 (181)	>2s	5.1 (1.8-14.1)	3 (1-6)	99 (99-100)	4.5 (1.6-12.8)	0.98 (0.95-1.00)
**Prediction of meningitis***									
McArdle, 2011[3]	ED, Ireland	Patients with meningococcal or pneumococcal PCR results	1825 (55)	delayed		71 (57-82)	95 (94-96)	15.1 (11.5-19.8)	0.31 (0.20-0.46)
Weber, 2003[4]	Tertiary hospitals, Ethiopia, the Gambia, Papua New Guinea, and the Philippines	Infants with a wide ranges of illness severity	3285 (34)	>2s	1.7 (0.8-3.4)	29 (15-47)	90 (89-91)	2.8 (1.7-4.8)	0.79 (0.63-0.98)
Wells, 2001[5]	Paediatric ED, UK	Infants and children presenting with non-blanching rash	217 (24)	>2s	29.4 (9.4-92.6)	83 (68-98)	85 (81-90)	5.7 (3.9-8.5)	0.19 (0.08-0.48)
**Severity of illness / infection**									
Thompson, 2009[6]	Paediatric assessment unit, UK	Children whose parents, referring clinician, or triage nurse suspected acute infection	392 (180)	>2s		8 (5-13)	99.5 (97-100)	17.7 (2.4-132.4)	0.92 (0.88-0.96)
Weber, 2003[4]	Tertiary hospitals, Ethiopia, the Gambia, Papua New Guinea, and the Philippines	Infants with a wide ranges of illness severity	3285 (372)	>2s	1.7 (0-6dys)<br>3.3 (7-60dys)	32 (27-37)	92 (91-93)	4.1 (3.4-4.9)	0.74 (0.69-0.79)
**Admission**									
Leonard, 2004[7]	Paediatric ED, UK	Children attending with recent onset of illness with no trauma	4878	>=3s		21 (19.2-22.9)	89 (88.3-90.5)	1.99	0.88
van den Bruel, 2007[8]	Primary care, Belgium	Children consulting with acute illness for	3981 (31)	>3s	42.96 (11.20-164.81)	10 .0 (0-20.74)	99.74 (99.58-99.9)	38.76 (11.23-133.83)	0.9 (0.8-1.02)

		maximum of five days							
YICSS Group, 2008[9]	Urban teaching hospitals, Bangladesh, Bolivia, Ghana, India, South Africa, and Pakistan	Infants attending with acute illness	8883 (1434)	prolonged	12.1 (0-6 dys) 31.6 (5-59dys)	4 (3-5)	99.7 (99.5-99.8)	13.1 (8.1-21.2)	0.96 (0.95-0.97)
**Sepsis, bacteraemia**									
Craig, 2010[10]	Paediatric ED, Australia	Children presenting with febrile illness	15781 (533)	>=2s	6.55				
Craig, 2010[10]	Paediatric ED, Australia	Children presenting with febrile illness	15781 (533)	>3s	38.7				
Verbakel, 2014*[11]	Hospital, UK	Children admitted from ED or after primary care referral	857 (50)	>=3s		25 (13.6-39.6)	97.1 (95.7-98.2)	8.66 (4.57-16.4)	0.77 (0.66-0.91)
Weber, 2003[4]	Tertiary hospitals, Ethiopia, the Gambia, Papua New Guinea, and the Philippines	Infants with a wide ranges of illness severity	3285 (119)	>2s	2.2 (1.5-3.3)	34 (25-43)	90 (89-91)	3.5 (2.6-4.5)	0.74 (0.65-0.84)
**Urinary tract infection**									
Craig, 2010[10]	Paediatric ED, Australia	Children presenting with febrile illness	15781 (543)	>=2s	2.16				
Craig, 2010[10]	Paediatric ED, Australia	Children presenting with febrile illness	15781 (543)	>3s	4.85				
**Pneumonia**									
Craig, 2010[10]	Paediatric ED, Australia	Children presenting with febrile illness	15781 (64)	>=2s	3.05				
Craig, 2010[10]	Paediatric ED, Australia	Children presenting with febrile illness	15781 (64)	>3s	2.62				
**Hypoxia**									
Weber, 2003[4]	Tertiary hospitals, Ethiopia, the Gambia, Papua New Guinea, and the Philippines	Infants with a wide ranges of illness severity	3176 (203)	>2s	2.7 (1.9-3.7)	34 (28-41)	91 (90-92)	3.8 (3.1-4.8)	0.72 (0.66-0.80)

* The outcome reported in Verbackel, 2014 is meningitis, bacteraemia, or sepsis. The study is grouped with bacteraemia and sepsis outcomes as there was a requirement in each case for a positive sterile site culture.

Reference List

1. Ahmed F, Mahmood C, Sharma J, Hoque S, Zaman R, et al. (2001) Dengue and dengue haemorrhagic fever in children during the 2000 outbreak in Chittagong, Bangladesh. Dengue Bulletin 25: 33-39.
2. Biswas HH, Ortega O, Gordon A, Standish K, Balmaseda A, et al. (2012) Early clinical features of dengue virus infection in nicaraguan children: a longitudinal analysis. PLoS Neglected Tropical Diseases [electronic resource] 6: e1562.
3. McArdle S, O'Sullivan R, Walsh S (2011) Utility of PCR testing for invasive meningococcal and pneumococcal disease in a paediatric emergency care setting. Academic Emergency Medicine Conference: 2011 Annual Meeting of the Society for Academic Emergency Medicine, SAEM Boston, MA United States. Conference Start: 20110601 Conference End: 20110605. Conference Publication:: S178.
4. Weber MW, Carlin JB, Gatchalian S, Lehmann D, Muhe L, et al. (2003) Predictors of neonatal sepsis in developing countries. Pediatr Infect Dis J 22: 711-717.
5. Wells LC, Smith JC, Weston VC, Collier J, Rutter N (2001) The child with a non-blanching rash: how likely is meningococcal disease? Archives of disease in childhood 85: 218-222.
6. Thompson M, Coad N, Harnden A, Mayon-White R, Perera R, et al. (2009) How well do vital signs identify children with serious infections in paediatric emergency care? Archives of disease in childhood 94: 888-893.
7. Leonard PA, Beattie TF (2004) Is measurement of capillary refill time useful as part of the initial assessment of children? European Journal of Emergency Medicine 11: 158-163.
8. Van den Bruel A, Aertgeerts B, Bruyninckx R, Aerts M, Buntinx F (2007) Signs and symptoms for diagnosis of serious infections in children: a prospective study in primary care. The British journal of general practice : the journal of the Royal College of General Practitioners 57: 538-546.
9. Young Infants Clinical Signs Study G (2008) Clinical signs that predict severe illness in children under age 2 months: a multicentre study. Lancet 371: 135-142.
10. Craig JC, Williams GJ, Jones M, Codarini M, Macaskill P, et al. (2010) The accuracy of clinical symptoms and signs for the diagnosis of serious bacterial infection in young febrile children: prospective cohort study of 15 781 febrile illnesses. BMJ 340: c1594.
11. Verbakel JY, MacFaul R, Aertgeerts B, Buntinx F, Thompson M (2014) Sepsis and meningitis in hospitalized children: performance of clinical signs and their prediction rules in a case-control study. Pediatric emergency care 30: 373-380.
