# Supplementary material for: The Diagnostic Value of Capillary Refill Time for Detecting Serious Illness in Children: A Systematic Review and Meta-Analysis
Source: PLoS One. 2015 Sep 16;10(9):e0138155. doi: 10.1371/journal.pone.0138155 (PMC4573516; doi:10.1371/journal.pone.0138155)
Supplement: S1 Text — (PDF) [file pone.0138155.s008.pdf]

Protocol for a systematic review of the diagnostic value of capillary refill time in children

NB: this is a companion review to a systematic review of the reliability and validity of capillary refill time in children

Change log

- Updated search strategy to more detailed methodology
- Removal of limitation to English language due to number of papers from LMIC settings identified
- Clarification of definition of "premature" and proportion of subjects with cardiorespiratory disease or prematurity before exclusion
- Clarification of inclusion if at least 20 non-excluded subjects (non-premature, under 18, and without cardiorespiratory disease) are separately reported
- Removal of laboratory results and provision of therapy alone as acceptable outcomes (based on clinical consensus)
- Clarification of procedure for numerical calculations during/after data extraction
- Updated search date (12 April 2013) added.
- Clarification of procedure for assessing HSROC heterogeneity
- Updated search date (9 June 2014) added.

Background

Capillary refill time (CRT) is used in primary and emergency care as part of the initial assessment of unwell children. It is included in a variety of national and international guidelines such as WHO guidelines for children with severe infection or malnutrition, and UK NICE guidelines for children with feverish illness, meningococcal disease, or gastroenteritis.[1-4]

Need for systematic review

While prolonged CRT or decreased peripheral perfusion are frequently cited as red flags for serious illness in children, there are currently no systematic reviews of the diagnostic accuracy of CRT to support these statements.

Research question(s)

- What is the diagnostic value of CRT to identify children with serious illness (e.g. dehydration, meningitis), and to predict adverse outcomes (e.g. death)?

Search strategy

Database search

A search strategy was developed in conjunction with an information specialist (N Roberts). Three bibliographic databases (Medline, Embase, and Cinahl) were searched using the strategies in Tables 1-3. These strategies were developed by refining an initial broad search strategy based on characteristics of useful papers identified.

	Search Terms
1	Capillary Resistance/
2	Capillaries/ph, pp [Physiology, Physiopathology]
3	Blood Flow Velocity/ph [Physiology]
4	(capillar* adj3 (refill* or fill*)).ti,ab.
5	(peripher* adj2 (perfus* or circulat* or shutdown)).ti,ab.
6	(perfusion adj2 time).ti,ab.
7	(skin adj2 turgor).ti,ab.

8	1 or 2 or 3 or 4 or 5 or 7
9	(Infan* or newborn* or new-born* or perinat* or neonat* or baby* or babies or toddler* or minors* or boy or boys or boyfriend or boyhood or girl* or kid or kids or child* or schoolchild* or school child* or adolescen* or juvenil* or youth* or teen* or under?age* or pubescen* or pediatric* or paediatric* or peadiatric* or school or schools or prematur* or preterm*).mp.
10	mortality/ or "cause of death"/ or child mortality/ or infant mortality/ or survival rate/
11	mo.fs.
12	(mortality or death? or survival).ti,ab.
13	10 or 11 or 12
14	8 and 9 and 13
15	sepsis/ or bacteremia/ or shock, septic/
16	Bacterial Infections/
17	meningitis/ or exp meningitis, bacterial/
18	exp meningococcal infections/
19	pneumonia/ or exp pneumonia, bacterial/
20	exp Urinary Tract Infections/
21	Gastroenteritis/
22	malaria/ or exp malaria, falciparum/
23	exp Dengue/
24	Shock/
25	Fever/
26	"Severity of Illness Index"/
27	infection?.ti.
28	(sepsis or septic or septicaemi* or septicemi* or bacteraemi* or bacteremi*).ti,ab.
29	((severe or severity or serious or bacteria*) adj3 infection?).ti,ab.
30	((severe or severity or serious) adj3 (illness* or condition?)).ti,ab.
31	(meningitis or meningococcal).ti,ab.
32	pneumonia.ti,ab.
33	((urin* adj2 infection*) or uti or utis).ti,ab.
34	gastroenteritis.ti,ab.
35	(fever* or febrile or shock).ti,ab.
36	Dehydration/
37	(dehydrat* or rehydrat*).ti,ab.
38	(fluid? adj2 (deficit? or deficienc* or imbalance?)).ti,ab.
39	(infection? adj2 (marker? or biomarker?)).ti,ab.
40	Anoxia/
41	(hypoxi? or anoxi? or (oxygen adj2 (deficien* or saturation))).ti,ab.
42	exp Leukocyte Count/
43	((white blood cell? or leukocyte?) adj2 (count? or test?)).ti,ab.
44	15 or 16 or 17 or 18 or 19 or 20 or 21 or 22 or 23 or 24 or 25 or 26 or 27 or 28 or 29 or 30 or 31 or 32 or 33 or 34 or 35 or 36 or 37 or 38 or 39 or 40 or 41 or 42 or 43
45	8 and 9 and 44
46	hospitalization/ or "length of stay"/
47	Patient Admission/
48	(hospitalisation? or hospitalization?).ti,ab.
49	"length of stay".ti,ab.
50	((hospital or patient?) adj2 stay*).ti,ab.
51	(patient adj2 (admission? or admitted)).ti,ab.
52	(hospital? adj2 (admission? or admitted)).ti,ab.
53	46 or 47 or 48 or 49 or 50 or 51 or 52
54	8 and 9 and 53

Table 1: Medline search strategy

	**Search Terms**

1	Capillary Resistance/
2	capillary flow/
3	(capillar* adj3 (refill* or fill*)).ti,ab.
4	(peripher* adj2 (perfus* or circulat* or shutdown)).ti,ab.
5	(perfusion adj2 time).ti,ab.
6	(skin adj2 turgor).ti,ab.
7	1 or 2 or 3 or 4 or 6
8	(Infan* or newborn* or new-born* or perinat* or neonat* or baby* or babies or toddler* or minors* or boy or boys or boyfriend or boyhood or girl* or kid or kids or child* or schoolchild* or school child* or adolescen* or juvenil* or youth* or teen* or under?age* or pubescen* or pediatric* or paediatric* or peadiatric* or school or schools or prematur* or preterm*).mp.
9	mortality/ or childhood mortality/ or infant mortality/ or death/ or "cause of death"/ or child death/ or survival rate/ or survival/
10	(mortality or death? or survival).ti,ab.
11	9 or 10
12	7 and 8 and 11
13	sepsis/ or exp bacteremia/ or septic shock/ or septicemia/
14	Bacterial Infection/
15	meningitis/ or bacterial meningitis/
16	exp meningococcosis/
17	pneumonia/ or exp infectious pneumonia/
18	exp urinary tract infection/
19	Gastroenteritis/
20	malaria/ or malaria falciparum/
21	Dengue/
22	Shock/
23	Fever/
24	disease severity/
25	infection?.ti.
26	(sepsis or septic or septicaemi* or septicemi* or bacteraemi* or bacteremi*).ti,ab.
27	((severe or severity or serious or bacteria*) adj3 infection?).ti,ab.
28	((severe or severity or serious) adj3 (illness* or condition?)).ti,ab.
29	(meningitis or meningococcal).ti,ab.
30	pneumonia.ti,ab.
31	((urin* adj2 infection*) or uti or utis).ti,ab.
32	gastroenteritis.ti,ab.
33	(fever* or febrile or shock).ti,ab.
34	Dehydration/
35	(dehydrat* or rehydrat*).ti,ab.
36	(fluid? adj2 (deficit? or deficienc* or imbalance?)).ti,ab.
37	(infection? adj2 (marker? or biomarker?)).ti,ab.
38	Anoxia/
39	(hypoxi? or anoxi? or (oxygen adj2 (deficien* or saturation))).ti,ab.
40	exp Leukocyte Count/
41	((white blood cell? or leukocyte?) adj2 (count? or test?)).ti,ab.
42	13 or 14 or 15 or 16 or 17 or 18 or 19 or 20 or 21 or 22 or 23 or 24 or 25 or 26 or 27 or 28 or 29 or 30 or 31 or 32 or 33 or 34 or 35 or 36 or 37 or 38 or 39 or 40 or 41
43	7 and 8 and 42
44	hospitalization/ or "length of stay"/ or child hospitalization/ or hospital utilization/
45	hospital admission/
46	(hospitalisation? or hospitalization?).ti,ab.
47	"length of stay".ti,ab.
48	((hospital or patient?) adj2 stay*).ti,ab.
49	(patient adj2 (admission? or admitted)).ti,ab.
50	(hospital? adj2 (admission? or admitted)).ti,ab.
51	44 or 45 or 46 or 47 or 48 or 49 or 50
52	7 and 8 and 51

Table 2: Embase search strategy

	Search terms
S1	(MH "Capillary Resistance")
S2	(MH "Capillaries/PH/PP")
S3	(MH "Blood Flow Velocity/PH")
S4	TI ((capillar* N3 (refill* or fill*))) OR AB ((capillar* N3 (refill* or fill*)))
S5	TI ((peripher* N2 (perfus* or circulat* or shutdown))) OR AB ((peripher* N2 (perfus* or circulat* or shutdown)))
S6	TI (perfusion N2 time) OR AB (perfusion N2 time)
S7	TI (skin N2 turgor) OR AB (skin N2 turgor)
S8	S1 OR S2 OR S3 OR S4 OR S5 OR S6 OR S7
S9	TI (Infan* or newborn* or new-born* or perinat* or neonat* or baby* or babies or toddler* or minors* or boy or boys or boyfriend or boyhood or girl* or kid or kids or child* or schoolchild* or school child* or adolescen* or juvenil* or youth* or teen* or under?age* or pubescen* or pediatric* or paediatric* or peadiatric* or school or schools or prematur* or preterm*) OR AB (Infan* or newborn* or new-born* or perinat* or neonat* or baby* or babies or toddler* or minors* or boy or boys or boyfriend or boyhood or girl* or kid or kids or child* or schoolchild* or school child* or adolescen* or juvenil* or youth* or teen* or under?age* or pubescen* or pediatric* or paediatric* or peadiatric* or school or schools or prematur* or preterm*) OR MW (Infan* or newborn* or new-born* or perinat* or neonat* or baby* or babies or toddler* or minors* or boy or boys or boyfriend or boyhood or girl* or kid or kids or child* or schoolchild* or school child* or adolescen* or juvenil* or youth* or teen* or under?age* or pubescen* or pediatric* or paediatric* or peadiatric* or school or schools or prematur* or preterm*)
S10	S8 AND S9

Table 3: Cinahl search strategy

Search date

The initial search date was 10 January 2013. Updated searches were performed on 12 April 2013 and 9 June 2014.

Additional searching

Additional papers were identified from the reference lists of relevant papers and consultation with experts.

Selection criteria

Inclusion criteria

- reports measurement of capillary refill time (by any manual method) on a minimum of 20 subjects under the age of 18 years
    - if a study includes a larger age range, separate reporting of results for subjects under the age of 18 years is required for inclusion
    - separate reporting of at least 20 subjects who were not born premature neonates and/or did not have pre-existing cardiorespiratory disease is also sufficient for inclusion
- Measure and report relationship between CRT and a relevant clinical outcome
    - mortality
    - final diagnosis of a specific serious illness (e.g. meningitis, dengue)
    - degree of dehydration / fluid deficit
    - admission to secondary care
    - severity of illness or outcome
    - hypoxaemia

Exclusion criteria

- subjects over the age of 18
- more than 50% of the subjects were neonates born prematurely (<35 weeks gestation)
- more than 50% of the subjects had significant pre-existing cardiorespiratory disease such as cardiac malformations

Selection of studies

- Screening – two authors screen against inclusion criteria, with disagreements resolved by consensus, any uncertain papers to be retained
- Final selection – full papers assessed against inclusion criteria by four authors

Quality assessment

Quality assessment criteria (Table 4) have been developed based on the QUADAS-2 guidelines.[5]

Patient selection	Bias: suitable sampling method used
	Bias: appropriate exclusion criteria
	Applicability: appropriate inclusion criteria
Index test (CRT)	Bias: blinded to result of comparator
	Bias: time measured OR pre-specified threshold used
	Applicability: site and time measurement method defined
Reference standard (clinical outcome)	Bias: blinded to index test result
	Bias: independent of result of index test
	Applicability: objective measurement
Timing and flow	Bias: contemporaneous measurement of index test and comparator
	Bias: all children had index test performed
	Bias: all children had comparator assessed

Table 4: Quality assessment criteria

Assessment is carried out by two authors (the same two authors to assess all papers), with disagreements resolved by consensus. Each criterion will be marked as "yes", "no" or "unclear". In the case of "Applicability: site and time measurement method defined", "yes" denotes that both are defined, with "unclear" used if only one (site or time measurement method) is defined.

Data extraction

The data extraction procedure was designed and piloted using a small number of studies. Data is extracted onto a Microsoft Excel spreadsheet, and double-checked for accuracy by a second author. Any disagreements should be resolved by consensus.

During data extraction, no additional calculations (e.g. completing 2x2 tables) should be carried out. Missing data should be left blank.

Where additional calculations are required prior to data analysis, this should be automated if possible (e.g. using a statistical program such as R). If this is not possible, calculations should be double-checked by a second author, and sanity-checking (e.g. checking of row and column sums in 2x2 tables) should be employed where possible.

Data analysis

Where appropriate (if there is no clinical heterogeneity and at least 4 studies), meta-analysis of results will be carried out using hierarchical summary ROC curves. Heterogeneity will be assessed visually by multiple authors, with determination of the presence and degree of heterogeneity by consensus.

If meta-analysis of the results is not appropriate, narrative description of the results will be employed. Tabulation of results or display using forest or dumbbell plots may be used to clarify the narrative description.

References

1 World Health Organization. Management of the child with a serious infection or severe malnutrition : guidelines for care at the first-referral level in developing countries. Geneva: Department of Child and Adolescent Health and Development UNICEF; 2000.

2 National Institute for Health and Care Excellence. Diarrhoea and vomiting caused by gastroenteritis: diagnosis, assessment and management in children younger than 5 years. CG84. London: National Institute for Health and Care Excellence; 2009.

3 National Institute for Health and Care Excellence. Bacterial meningitis and meningococcal septicaemia. CG102. London: National Institute for Health and Care Excellence; 2010.

4 National Institute for Health and Care Excellence. Feverish illness in children. CG160. London: National Institute for Health and Care Excellence; 2013.

5 Whiting PF, Rutjes AW, Westwood ME, et al. QUADAS-2: a revised tool for the quality assessment of diagnostic accuracy studies. Ann Intern Med 2011; 155(8): 529–36.
