# Supplementary material for: The Diagnostic Value of Capillary Refill Time for Detecting Serious Illness in Children: A Systematic Review and Meta-Analysis
Source: PLoS One. 2015 Sep 16;10(9):e0138155. doi: 10.1371/journal.pone.0138155 (PMC4573516; doi:10.1371/journal.pone.0138155)
Supplement: S2 Text — (PDF) [file pone.0138155.s009.pdf]

S2 Text: Search strategy for diagnostic accuracy studies
The search date for all searches was 9 June 2014.

Medline (OvidSP) [1946 – current, In process] search strategy

	Search Terms
1	Capillary Resistance/
2	Capillaries/ph, pp [Physiology, Physiopathology]
3	Blood Flow Velocity/ph [Physiology]
4	(capillar* adj3 (refill* or fill*)).ti,ab.
5	(peripher* adj2 (perfus* or circulat* or shutdown)).ti,ab.
6	(perfusion adj2 time).ti,ab.
7	(skin adj2 turgor).ti,ab.
8	1 or 2 or 3 or 4 or 5 or 7
9	(Infan* or newborn* or new-born* or perinat* or neonat* or baby* or babies or toddler* or minors* or boy or boys or boyfriend or boyhood or girl* or kid or kids or child* or schoolchild* or school child* or adolescen* or juvenil* or youth* or teen* or under?age* or pubescen* or pediatric* or paediatric* or peadiatric* or school or schools or prematur* or preterm*).mp.
10	mortality/ or "cause of death"/ or child mortality/ or infant mortality/ or survival rate/
11	mo.fs.
12	(mortality or death? or survival).ti,ab.
13	10 or 11 or 12
14	8 and 9 and 13
15	sepsis/ or bacteremia/ or shock, septic/
16	Bacterial Infections/
17	meningitis/ or exp meningitis, bacterial/
18	exp meningococcal infections/
19	pneumonia/ or exp pneumonia, bacterial/
20	exp Urinary Tract Infections/
21	Gastroenteritis/
22	malaria/ or exp malaria, falciparum/
23	exp Dengue/
24	Shock/
25	Fever/
26	"Severity of Illness Index"/
27	infection?.ti.
28	(sepsis or septic or septicaemi* or septicemi* or bacteraemi* or bacteremi*).ti,ab.
29	((severe or severity or serious or bacteria*) adj3 infection?).ti,ab.
30	((severe or severity or serious) adj3 (illness* or condition?)).ti,ab.
31	(meningitis or meningococcal).ti,ab.
32	pneumonia.ti,ab.
33	((urin* adj2 infection*) or uti or utis).ti,ab.
34	gastroenteritis.ti,ab.
35	(fever* or febrile or shock).ti,ab.
36	Dehydration/
37	(dehydrat* or rehydrat*).ti,ab.
38	(fluid? adj2 (deficit? or deficienc* or imbalance?)).ti,ab.
39	(infection? adj2 (marker? or biomarker?)).ti,ab.
40	Anoxia/
41	(hypoxi? or anoxi? or (oxygen adj2 (deficien* or saturation))).ti,ab.
42	exp Leukocyte Count/
43	((white blood cell? or leukocyte?) adj2 (count? or test?)).ti,ab.
44	15 or 16 or 17 or 18 or 19 or 20 or 21 or 22 or 23 or 24 or 25 or 26 or 27 or 28 or 29 or 30 or 31 or 32 or 33 or 34 or 35 or 36 or 37 or 38 or 39 or 40 or 41 or 42 or 43
45	8 and 9 and 44
46	hospitalization/ or "length of stay"/
47	Patient Admission/

48	(hospitalisation? or hospitalization?).ti,ab.
49	"length of stay".ti,ab.
50	((hospital or patient?) adj2 stay*).ti,ab.
51	(patient adj2 (admission? or admitted)).ti,ab.
52	(hospital? adj2 (admission? or admitted)).ti,ab.
53	46 or 47 or 48 or 49 or 50 or 51 or 52
54	8 and 9 and 53

Embase (Ovid SP) [1974 – current] search strategy

	**Search Terms**
1	Capillary Resistance/
2	capillary flow/
3	(capillar* adj3 (refill* or fill*)).ti,ab.
4	(peripher* adj2 (perfus* or circulat* or shutdown)).ti,ab.
5	(perfusion adj2 time).ti,ab.
6	(skin adj2 turgor).ti,ab.
7	1 or 2 or 3 or 4 or 6
8	(Infan* or newborn* or new-born* or perinat* or neonat* or baby* or babies or toddler* or minors* or boy or boys or boyfriend or boyhood or girl* or kid or kids or child* or schoolchild* or school child* or adolescen* or juvenil* or youth* or teen* or under?age* or pubescen* or pediatric* or paediatric* or peadiatric* or school or schools or prematur* or preterm*).mp.
9	mortality/ or childhood mortality/ or infant mortality/ or death/ or "cause of death"/ or child death/ or survival rate/ or survival/
10	(mortality or death? or survival).ti,ab.
11	9 or 10
12	7 and 8 and 11
13	sepsis/ or exp bacteremia/ or septic shock/ or septicemia/
14	Bacterial Infection/
15	meningitis/ or bacterial meningitis/
16	exp meningococcosis/
17	pneumonia/ or exp infectious pneumonia/
18	exp urinary tract infection/
19	Gastroenteritis/
20	malaria/ or malaria falciparum/
21	Dengue/
22	Shock/
23	Fever/
24	disease severity/
25	infection?.ti.
26	(sepsis or septic or septicaemi* or septicemi* or bacteraemi* or bacteremi*).ti,ab.
27	((severe or severity or serious or bacteria*) adj3 infection?).ti,ab.
28	((severe or severity or serious) adj3 (illness* or condition?)).ti,ab.
29	(meningitis or meningococcal).ti,ab.
30	pneumonia.ti,ab.
31	((urin* adj2 infection*) or uti or utis).ti,ab.
32	gastroenteritis.ti,ab.
33	(fever* or febrile or shock).ti,ab.
34	Dehydration/
35	(dehydrat* or rehydrat*).ti,ab.
36	(fluid? adj2 (deficit? or deficienc* or imbalance?)).ti,ab.
37	(infection? adj2 (marker? or biomarker?)).ti,ab.
38	Anoxia/
39	(hypoxi? or anoxi? or (oxygen adj2 (deficien* or saturation))).ti,ab.

40	exp Leukocyte Count/
41	((white blood cell? or leukocyte?) adj2 (count? or test?)).ti,ab.
42	13 or 14 or 15 or 16 or 17 or 18 or 19 or 20 or 21 or 22 or 23 or 24 or 25 or 26 or 27 or 28 or 29 or 30 or 31 or 32 or 33 or 34 or 35 or 36 or 37 or 38 or 39 or 40 or 41
43	7 and 8 and 42
44	hospitalization/ or "length of stay"/ or child hospitalization/ or hospital utilization/
45	hospital admission/
46	(hospitalisation? or hospitalization?).ti,ab.
47	"length of stay".ti,ab.
48	((hospital or patient?) adj2 stay*).ti,ab.
49	(patient adj2 (admission? or admitted)).ti,ab.
50	(hospital? adj2 (admission? or admitted)).ti,ab.
51	44 or 45 or 46 or 47 or 48 or 49 or 50
52	7 and 8 and 51

CINAHL (EBSCOHost) [1980 – current] search strategy

	Search terms
S1	(MH "Capillary Resistance")
S2	(MH "Capillaries/PH/PP")
S3	(MH "Blood Flow Velocity/PH")
S4	TI ((capillar* N3 (refill* or fill*))) OR AB ((capillar* N3 (refill* or fill*)))
S5	TI ((peripher* N2 (perfus* or circulat* or shutdown))) OR AB ((peripher* N2 (perfus* or circulat* or shutdown)))
S6	TI (perfusion N2 time) OR AB (perfusion N2 time)
S7	TI (skin N2 turgor) OR AB (skin N2 turgor)
S8	S1 OR S2 OR S3 OR S4 OR S5 OR S6 OR S7
S9	TI (Infan* or newborn* or new-born* or perinat* or neonat* or baby* or babies or toddler* or minors* or boy or boys or boyfriend or boyhood or girl* or kid or kids or child* or schoolchild* or school child* or adolescen* or juvenil* or youth* or teen* or under?age* or pubescen* or pediatric* or paediatric* or peadiatric* or school or schools or prematur* or preterm*) OR AB (Infan* or newborn* or new-born* or perinat* or neonat* or baby* or babies or toddler* or minors* or boy or boys or boyfriend or boyhood or girl* or kid or kids or child* or schoolchild* or school child* or adolescen* or juvenil* or youth* or teen* or under?age* or pubescen* or pediatric* or paediatric* or peadiatric* or school or schools or prematur* or preterm*) OR MW (Infan* or newborn* or new-born* or perinat* or neonat* or baby* or babies or toddler* or minors* or boy or boys or boyfriend or boyhood or girl* or kid or kids or child* or schoolchild* or school child* or adolescen* or juvenil* or youth* or teen* or under?age* or pubescen* or pediatric* or paediatric* or peadiatric* or school or schools or prematur* or preterm*)
S10	S8 AND S9
